# **Morpho-molecular characterization of** ***Trichodina chlorophora*** **Richards, 1948 (Protista: Ciliophora), a central component in the ‘snail‒ciliate‒zoochlorellae’ hyper-symbiotic system**

**DOI:** 10.1007/s42995-026-00359-4

**Published:** 2026-04-07

**Authors:** Tengyue Zhang, Peter Vďačný

**Affiliations:** 1 The Key Laboratory of Zoological Systematics and Application, College of Life Sciences, Hebei University, Baoding, 071002 China; 2 Department of Zoology, Faculty of Natural Sciences, Comenius University in Bratislava, 842 15 Bratislava, Slovak Republic

**Keywords:** *Chlorella*, Coevolution, Endosymbionts, Integrative taxonomy, *Physella*, Tripartite symbiotic consortium

## Abstract

In the mantle cavity of the heterobranch snail *Physella acuta*, collected from a lake in Slovakia (Central Europe), we identified the peritrich ciliate *Trichodina chlorophora* harboring endosymbiotic green algae. To elucidate the evolutionary origins of this tripartite consortium, we determined the phylogenetic affiliations of all three partners and conducted a detailed morpho-molecular characterization of the ciliate, a central component of this hyper-symbiotic system. The European population of *T. chlorophora* closely matched North American populations previously described from physinine snails. The diagnostic features of *T. chlorophora* include: body diameter of 41–83 μm after dry silver nitrate impregnation; denticle ring 23–39 μm wide, with 23–30 denticles and 9–11 radial pins per denticle; denticles 5.7–7.8 μm long; adoral ciliary spiral performing ~ 1.13 turns (390°–409°) around peristomial disc; and a horseshoe-shaped macronucleus. Phylogenetic analyses revealed that: (1) the host snails are closely related to North American conspecifics, reflecting the human-mediated introduction of this invasive gastropod to Europe; (2) trichodinids colonized aquatic snails multiple times independently from poikilothermic vertebrate hosts, with *T. chlorophora* clustering with freshwater congeners from frogs, snails, and planarians; and (3) the endosymbiotic green algae comprise two species: *Chlorella* sp., closely related to endosymbionts of heliozoans and cnidarians, and *Jaagichlorella geometrica*, which clusters with epiphytic congeners. While the algae exhibit low host specificity, snail-dwelling *Trichodina* species show high phylogenetic host specificity. The parallel emergence of green algae-bearing trichodinids in physinine and planorbid snails suggests co-evolutionary processes that independently gave rise to interdependent associations among aquatic snails, ciliates, and zoochlorellae.

**Supplementary Information:**

The online version contains supplementary material available at 10.1007/s42995-026-00359-4.

## Introduction

Symbioses encompass a wide range of interspecies interactions, from mutually beneficial relationships (mutualism) to those where one species is disadvantaged (amensalism). These interactions are recognized as major drivers of ecological dynamics and evolutionary innovation, fostering interdependence and coevolution among diverse organisms (Drew et al. 2021; Song et al. 2024; Zilber-Rosenberg and Rosenberg 2008). Among eukaryotes, ciliates are particularly notable for their diverse symbiotic associations, with prokaryotes (bacteria and archaea), algae (zoochlorellae and zooxanthellae), protists, and animals, making them valuable models for studying evolution, biodiversity, and metabolic integration of symbiotic systems (for reviews, see Dagar et al. (2024); Song et al. (2024)).

Endozoic ciliates themselves often harbor a variety of endosymbiotic bacteria and archaea (e.g., Sauvadet et al. 2017; Vďačný et al. 2018; Williams and Coleman 1992). While several unrelated free-living ciliates have independently evolved stable, mutualistic relationships with algal symbionts (Dagar et al. 2024; Song et al. 2024), complex hyper-symbiotic systems involving animals, ciliates, and green algae (zoochlorellae) are rare. One such tripartite consortium was first described by Richards (1948) in North American physinine snails (subfamily Physinae), where trichodinid ciliates host endosymbiotic algae within the snail’s mantle cavity. Similar systems evolved independently not only in Central European planorbid snails (family Planorbidae) (Raabe 1965; Raabe and Raabe 1961), but also in various freshwater snails in the Americas (Blecka and Garoian 1972; Bovee 1961; Hertel et al. 2004; Machado Filho 1965; Richards 1948) and Asia (Wiroonpan and Purivirojkul 2019). Approximately one-third of *Trichodina* species inhabiting snail mantle cavities carry zoochlorellae, suggesting co-evolutionary processes that independently gave rise to interdependent associations among aquatic snails, ciliates, and zoochlorellae.

In the mantle cavity of the heterobranch snail *Physella acuta*, collected from a lake in Central Europe, we identified the peritrich ciliate *Trichodina chlorophora* harboring endosymbiotic green algae. This globally invasive aquatic snail was introduced into southern Europe in the eighteenth century and reached Central Europe by the mid-twentieth century (Horsák et al. 2013). Accurate morphological identification combined with molecular taxonomy is essential for understanding the evolution of symbiotic systems and their geographic distribution. Morphological traits provide critical diagnostic features for species delimitation and ecological interpretation, while molecular data offer robust phylogenetic frameworks that reveal evolutionary relationships and patterns of host specificity (Obert et al. 2025; Pecina et al. 2025; Rataj and Vďačný 2021; Zhang and Vďačný 2024; Zhang et al. 2023). This integrative approach is particularly important in complex symbioses, where convergent evolution and cryptic diversity may obscure true affiliations. For these reasons, we conducted a detailed morpho-molecular characterization of *T. chlorophora*, the central component of the ‘snail–ciliate–zoochlorellae’ hyper-symbiotic system, to clarify its taxonomic identity and phylogenetic position.

**Fig. 1:**
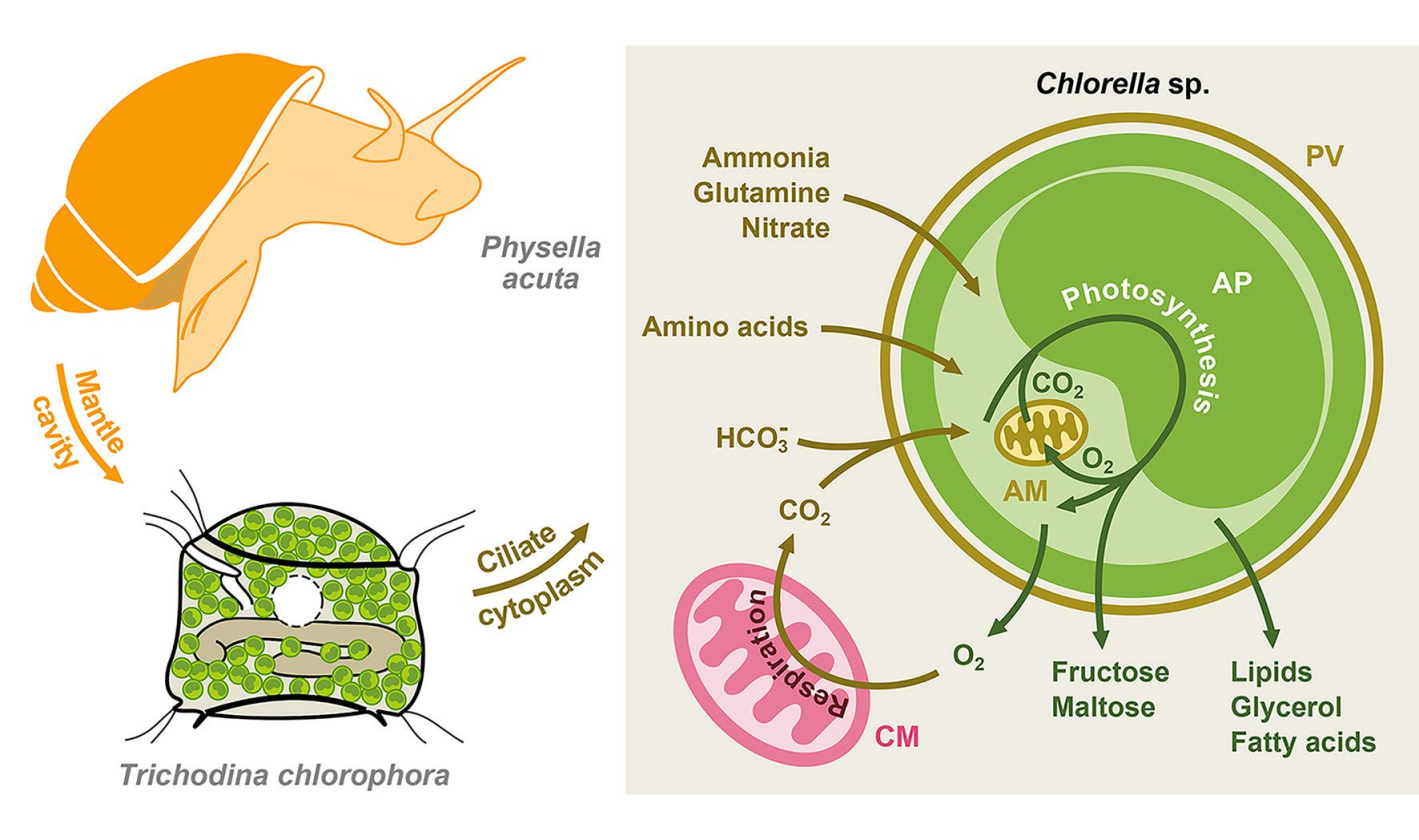
Schematic representation of the *Physella acuta*–*Trichodina chlorophora*–*Chlorella* sp. symbiotic system, illustrating the main predicted metabolic interactions between *T. chlorophora* and *Chlorella* sp. (based on Song et al. 2024). The snail provides shelter for both the trichodinid ciliates and their endosymbiotic green algae as well as protection from potential competitors and viral infections. Brown arrows indicate the translocation of CO_2_, carbon and nitrogen sources, and amino acids into *Chlorella* cells. Green arrows indicate the release of O_2_ and organic matter by endosymbiotic *Chlorella* cells for host cell utilization. *AM* algal mitochondrion, *AP* algal plastid, *CM* ciliate mitochondrion, *PV* perialgal vacuole

The low phylogenetic host specificity of the genus *Trichodina* (Zhang et al. 2023) and of endosymbiotic green algae (e.g., Hoshina et al. 2005; Kawaida et al. 2013; Kreutz et al. 2012; Pitsch et al. 2017; Pröschold et al. 2011) suggests that co-evolutionary mechanisms may have independently shaped these interdependent associations. Snails provide a protective habitat for both ciliates and their algal symbionts, shielding them from competitors and potential infections, such as those caused by *Chlorella* viruses (Iwai et al. 2019; Yamada et al. 2006). The translucent shells of physinine and planorbid snails allow sufficient light penetration, enabling photosynthesis by the endosymbiotic algae residing in the ciliate cytoplasm. These algae benefit from inorganic nutrients (e.g., nitrogen and CO_2_), derived from the metabolism of both the ciliate and the snail, and, in return, supply oxygen and maltose (Fig. 1; Kato et al. 2006; Song et al. 2024; Sørensen et al. 2020). This metabolic exchange allows ciliates to reduce their dependence on external food sources. Nevertheless, their well-developed oral ciliature, as shown in this study, enables them to continue filtering tissue debris, mucus, bacteria, and other microorganisms from the snail’s mantle cavity.

The evolutionary origins of these complex symbioses between snails, heterotrophic ciliates, and photosynthetic algae remain unclear. In this study, we examined the ciliate component of this hyper-symbiotic system and determined the phylogenetic affiliations of all three partners. Our aim was to test the following hypotheses: (1) trichodinids colonized aquatic snails multiple times independently from poikilothermic vertebrate hosts (Zhang et al. 2023); (2) trichodinids associated with aquatic snails exhibit broad geographic ranges and host spectra (Blecka and Garoian 1972); and (3) *Chlorella* and *Chlorella*-like algae display low phylogenetic host specificity, supporting their promiscuity across diverse hosts (Pröschold et al. 2011).

## Materials and methods

### Sampling and material processing

Nine specimens of the freshwater snail *Physella acuta* (Draparnaud, 1805) were collected from the Vojčianske jazero (lake), Vojka nad Dunajom, southwestern Slovakia (47°57′16″ N, 17°24′34″ E), to examine their mantle cavities for the presence of trichodinid ciliates. Snail identification was based on Horsák et al. (2013) and DNA barcoding of the mitochondrial cytochrome *c* oxidase subunit I gene (COI). The PCR procedure followed the protocol described by Zhang et al. (2023).

Trichodinid ciliates were studied in vivo and after dry silver nitrate (Klein 1958) and protargol (Wilbert 1975) impregnation. Silver nitrate impregnation was used to visualize the morphological characteristics of the adhesive disc, while protargol impregnation revealed the nuclear apparatus, oral ciliary pattern, and fine structure of the aboral ciliary wreath. General terminology and measurements for trichodinids follow Zhang et al. (2023), while the terminology for oral ciliature is based on Lynn (2008).

Trichodinids were washed to remove host-derived material and then sampled for molecular analyses. In total, 13 samples were prepared, each containing a single cell placed in 180 μL of cell lysis buffer (Cat. no. A1732, Promega, Fitchburg, WI, USA). These ciliate samples were also used for the molecular characterization of their endosymbiotic algae.

### Molecular methods

DNA extraction was performed with the ReliaPrep™ Blood gDNA Miniprep System (Cat. no. A5081, Promega, Fitchburg, WI, USA). PCR amplification was carried out with the GoTaq^®^ Long PCR Master Mix (Cat. no. M4021, Promega, Fitchburg, WI, USA). For trichodinids, two mitochondrial markers (16S rRNA gene and cytochrome *c* oxidase subunit I) and five nuclear markers (18S rRNA gene, internal transcribed spacer I (ITS1), 5.8S rRNA gene, internal transcribed spacer II (ITS2), and the first barcoding domain (D1) of the 28S rRNA gene) were amplified following the protocols of Rataj and Vďačný (2021). Zoochlorellae-target PCRs were performed with two sets of primers. The primer pair SR-1 (5′-TAC CTG GTT GAT CCT GCC AG-3′) and INT-5R (5′-AGG TGG GAG GGT TTA ATG AA-3′) (Hoshina et al. 2004) was used to amplify the nuclear 18S rRNA gene. The second pair, INT-4F (5′-TGG TGA AGT GTT CGG ATT GG-3′) (Hoshina et al. 2004) and HLR3R (5′-TCC CAA ACA ACC CGA CTC T-3′) (Hoshina et al. 2005), was used to amplify the nuclear ITS1-5.8S-ITS2-28S fragment. PCR conditions were identical to those used for trichodinids.

The quality of PCR products was verified by agarose gel electrophoresis. Products were purified with EPPiC Fast (A&A Biotechnology, Gdańsk, Poland) and bidirectionally Sanger sequenced on an ABI 3730 automatic sequencer (Macrogen Europe B.V., Amsterdam, The Netherlands).

### Phylogenetic methods

To determine the phylogenetic position of the obtained trichodinid sequences within the order Mobilida, two datasets were assembled. The first dataset contained 18S rRNA gene sequences, with taxon sampling following Zhang et al. (2023) (number of taxa = 65; number of characters = 1680). The second dataset comprised sequences from two mitochondrial markers (16S and COI) and five nuclear markers (18S, ITS1, 5.8S, ITS2, and D1-28S); only mobilids with all seven markers available were included (number of taxa = 52; number of characters = 4171). Two additional datasets were prepared to determine the phylogenetic positions of the zoochlorellae within the class Trebouxiophyceae. One dataset included only 18S sequences (number of taxa = 130; number of characters = 5738), while the other combined 18S with ITS1-5.8S-ITS2-28S sequences (number of taxa = 114; number of characters = 7667). Introns within algal rRNA genes were retained. GenBank accession numbers for all sequences used in datasets 1‒4 are provided in Supplementary Tables S1‒S3.

Individual molecular markers were aligned using MAFFT ver. 7 (Katoh et al. 2019) with the E-INS-i strategy, the 200PAM/κ = 2 scoring matrix, and a gap opening penalty of 1.53. No masking was applied. Maximum likelihood (ML) trees were constructed using IQ-TREE ver. 1.6.12 (Nguyen et al. 2015) via the IQ-TREE web server (http://iqtree.cibiv.univie.ac.at/) (Trifinopoulos et al. 2016). The best-fit substitution model for each marker was selected using ModelFinder with Bayesian Information Criterion (Kalyaanamoorthy et al. 2017), restricted to models compatible with MrBayes using the -mset option. Nodal support was assessed with 1000 ultrafast bootstrap pseudoreplicates, using the bnni algorithm to avoid overestimation (Hoang et al. 2018). Bayesian inference was conducted with MrBayes on XSEDE ver. 3.2.7a (Ronquist et al. 2012) via the CIPRES portal (http://www.phylo.org/) (Miller et al. 2010). Model parameters were specified using the ‘lset’ and ‘prset’ commands (Supplementary Table S4), allowing control over the number of substitution types (nst), substitution rate priors (revmatpr), and base frequency settings (statefreqpr). Two independent MCMC runs were performed, each with five million generations, sampling every 100 generations and discarding the first 25% as burn-in. Convergence to stationarity was assessed as described in Zhang et al. (2023). Resulting trees were visualized using FigTree ver. 1.2.3 (http://tree.bio.ed.ac.uk/software/figtree/).

The secondary structures of the 18S and 5.8S rRNA molecules and of the D1 domain of the 28S rRNA molecule were modeled with R2DT (Sweeney et al. 2021), following the procedure described by Zhang et al. (2023). Helices were numbered according to Petrov et al. (2014). The putative secondary structure of the ITS2 molecule was estimated using the free-energy minimization approach and the homology modeling via the Mfold web server ver. 3.0 (http://www.unafold.org/) (Zuker 2003). Folding of the ITS2 molecule was constrained according to the trichodinid model proposed by Rataj and Vďačný (2021). The resulting secondary structures were visualized using Traveller (Elias and Hoksza 2017) and VARNA ver. 3.93 (Darty et al. 2009). Three-dimensional models were predicted using RNAComposer ver. 1.0 (http://rnacomposer.cs.put.poznan.pl/) (Popenda et al. 2012).

## Results

### ***Trichodina chlorophora*** Richards, 1948 (Figs. 2, 3; Table 1)

**Fig. 2:**
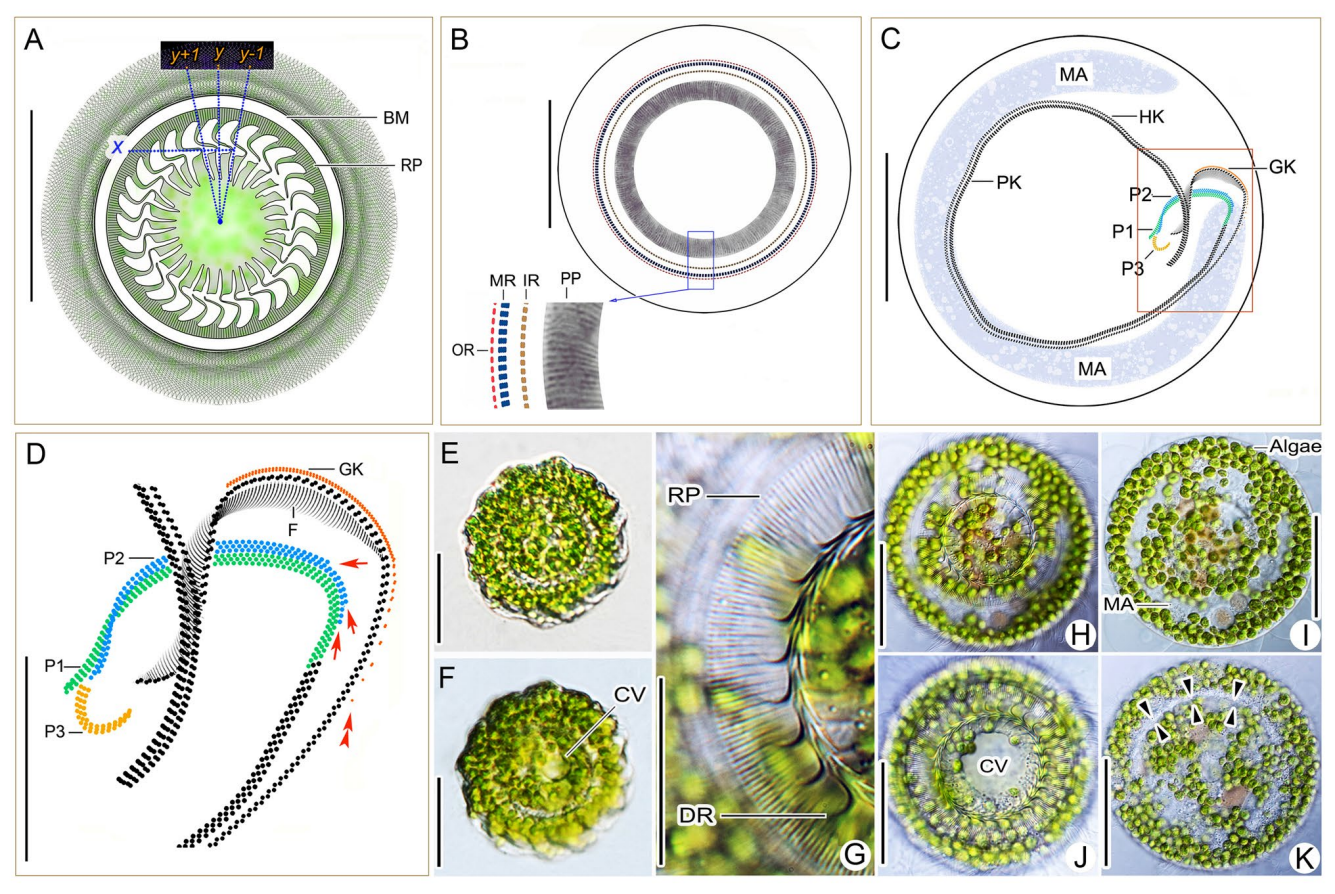
*Trichodina chlorophora* after dry silver nitrate (**A**), protargol (**B–D**) impregnation, and in vivo (**E–K**). **A** Overview of the aboral side, showing the structure of the adhesive disc. **B** Overview and fine structure of the aboral ciliary wreath. **C** Overview of the oral side, showing the oral ciliary pattern and macronucleus. **D** Detail of the oral ciliary pattern. Arrows mark the gradually shortened rows of dikinetids at the distal end of peniculus 2, and the double-arrowhead marks the widely spaced dikinetids forming the tail of the germinative kinety. **E, F** Overview of swimming individuals. **G** Detail of the denticle ring and radial pins. **H, J** Overviews of the aboral side. **I, K** Overviews of the oral side, showing the C-shaped macronucleus, endosymbiotic green algae, and adoral ciliary spiral (opposed triangles). *BM* border membrane, *CV* contractile vacuole, *DR* denticle ring, *F* oral fibers, *GK* germinative kinety, *HK* haplokinety, *IR* inner ring, *MA* macronucleus, *MR* middle ring, *OR* outer ring, *P1–3* peniculus 1–3, *PK* polykinety, *PP* peripheral pins; *RP* radial pins. Scale bars: 15 μm (**D, G**), 40 μm (**A–C, E, F, H–K**)

**Fig. 3:**
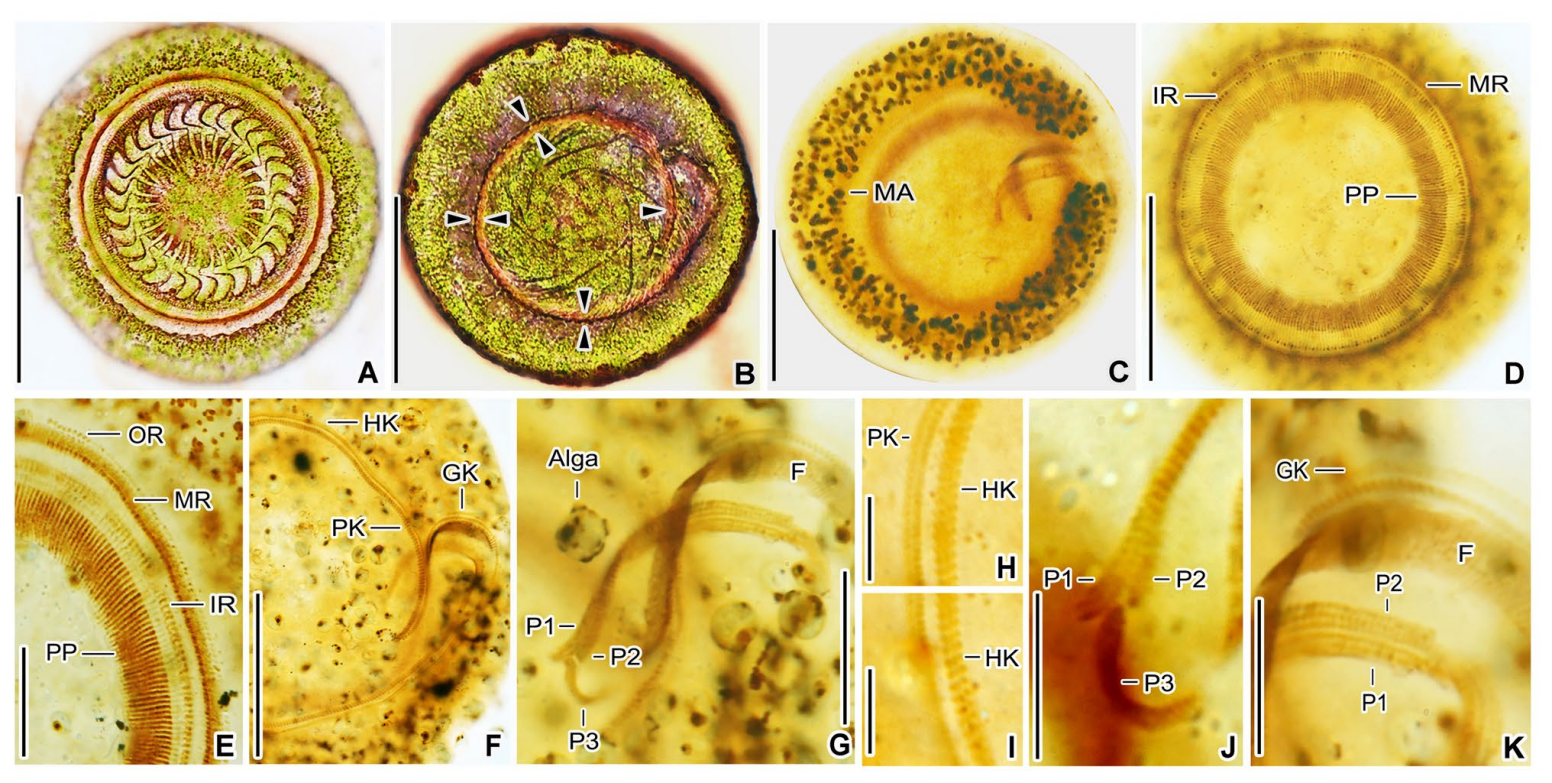
*Trichodina chlorophora* after dry silver nitrate (**A, B**) and protargol (**C–K**) impregnation. **A** Overview of the adhesive disc. **B** Overview of the oral side, showing the adoral ciliary spiral (opposed triangles). **C** Overview of the C-shaped macronucleus. **D, E** Aboral view, showing the structure of the ciliary wreath and peripheral pins. **F–K** Details of the oral ciliary pattern. *F* oral fibers, *GK* germinative kinety, *HK* haplokinety, *IR* inner ring, *MA* macronucleus, *MR* middle ring, *OR* outer ring, *P1–3* peniculus 1–3, *PK* polykinety, *PP* peripheral pins. Scale bars: 3 μm (**H, I**), 8 μm (**J, K**), 15 μm (**E, G**), 40 μm (**A–D, F**)

**Table 1:** Morphometric data on the Slovak population of *Trichodina chlorophora* isolated from *Physella acuta*

Character	Mean	M	SD	SE	CV	Min	Max	*n*
Body, diameter	50.2	50.2	2.7	0.7	5.5	45.7	54.7	15
Adhesive disc, diameter	45.2	44.6	2.4	0.6	5.4	41.3	48.6	15
Border membrane, width	2.5	2.4	0.4	0.1	15.9	1.9	3.1	15
Exring, diameter	40.1	40.1	2.6	0.7	6.5	36.7	43.9	15
Denticle ring, diameter	28.2	27.9	1.6	0.4	5.5	26.2	30.8	15
Central zone, diameter	17.8	17.5	1.2	0.3	6.5	16.2	19.9	15
Denticle, number	27.5	28.0	1.4	0.4	5.1	26.0	30.0	15
Denticle, length	6.5	6.2	0.7	0.2	10.7	5.7	7.8	15
Denticle, span	11.6	11.7	1.3	0.3	10.8	8.7	13.1	15
Ray, length	4.8	4.7	0.8	0.2	17.5	3.1	6.2	15
Central part of denticle, width	1.6	1.5	0.5	0.1	30.0	1.0	2.7	15
Blade, length	5.2	5.2	0.3	0.1	5.9	4.6	5.8	15
Blade, width	2.8	2.8	0.3	0.1	10.8	2.3	3.2	14
Radial pins per denticle, number	10.1	10.0	0.5	0.1	4.5	9.0	11.0	15
Radial pins, total number	276.9	280.0	14.7	3.8	5.3	252.0	300.0	15
Blade connection, width	0.6	0.6	0.2	0.0	23.5	0.4	0.9	15
Ray connection, width	0.9	0.8	0.2	0.1	25.9	0.4	1.2	15
Denticle above *x* axis, length	6.0	6.3	0.5	0.1	8.7	4.9	6.6	15
Denticle below *x* axis, length	5.5	5.9	0.9	0.2	16.0	3.8	6.6	15
Ratio of denticle length above and below *x* axis	1.1	1.1	0.2	0.0	14.6	0.9	1.4	15

Data based on randomly selected specimens after silver nitrate impregnation. All measurements are in μm

*CV* coefficient of variation in %, *M* median, *Max* maximum, *Mean* arithmetic mean, *Min* minimum, *n* number of individuals investigated, *SD* standard deviation, *SE* standard error of arithmetic mean

#### Nomenclature

*Trichodina chlorophora* was established in Richards’ doctoral thesis in 1948. However, the validity of names published in theses requires careful evaluation to determine whether they meet the criteria for publication under the International Code of Zoological Nomenclature (ICZN 1999). According to Article 8, a work is considered published if it constitutes a public and permanent scientific record, is obtainable, and exists in numerous identical, durable copies. Although dissertations are not explicitly excluded under Article 9.9 of the ICZN (1999), they typically do not meet these criteria (Aescht 2001; Berger 2006, p. 498).

Nevertheless, we accept Richards (1948) as the original description of *T. chlorophora* for the following reasons: (1) the dissertation was published in hard copy and is publicly available at Stanford Libraries (https://searchworks.stanford.edu/view/2161899); (2) Richards (1948) properly labeled type material (hypotype #71926), deposited it in an internationally recognized institution (U.S. National Museum Helminthological Collection), and provided a sufficient morphological description; (3) he specified the type locality (ponds near Stanford University, southern California, USA) and the type host (*Physa ampullacea* A. Gould, 1865, now considered a junior synonym of *Physella gyrina* (Say, 1821) according to MolluscaBase eds. 2025); (4) Blecka and Garoian (1972) revised the species and acknowledged Richards (1948) as the original author; and (5) Richards’ (1948) thesis has been cited multiple times, albeit with inconsistencies in dating (e.g., Blecka and Garoian 1972; Bovee 1961; Hertel et al. 2004).

### Improved diagnosis (based on Slovak and North American populations)

Medium-sized freshwater trichodinid with a body diameter of 41–83 (55) μm after dry silver nitrate impregnation. Adhesive disc 36–65 (45) μm in diameter, surrounded by a finely striated border membrane 1.5–3.1 (2.3) μm wide. Denticle ring 23–39 (30) μm in diameter, composed of 23–30 (27) denticles, each with 9–11 radial pins. Denticles 5.7–7.8 (7.0) μm long. Ratio of denticle length above and below the *x* axis 0.9–1.4 (1.1). Blade length 4.0–7.0 (5.1) μm; ray length 2.0–7.0 (4.7) μm; central part width 1.0–2.7 (1.6) μm. Blade sickle-shaped, broad, occupying approximately two-thirds of space between *y* axes. Anterior blade margin extends slightly beyond the *y* + 1 axis. Distal blade margin rounded to slightly truncated. Tangent point distinct, located at or below the level of the distal blade surface. Adoral ciliary spiral ca. 390°–409° (401°). Macronucleus C- or horseshoe-shaped, about 55 μm in diameter, 10 μm wide, and 140 μm long in the “uncoiled” condition after protargol impregnation. Micronucleus 3.0–4.5 × 3.5–5.3 μm, located in the + *y* position, + *y* value = 4‒14 (8). Zoochlorellae present.

### Etymology

The species-group name *chlorophora* is derived from the Greek adjective *χλωρός* (*khlōrós*, pale green), the thematic vowel ·*o*-, and the Latin suffix -*phor*·*us*, -*a*, -*um* [m, f, n] (bearing), referring to the pale green coloration of the ciliate caused by its zoochlorellae.

### Description of a Slovak population

The body outline is disc-shaped (rounded) when the ciliate is observed from the oral side (Figs. 2A, B, H, J, 3A, D), aboral side (Figs. 2C, 3B), and in optical sections through the mid-body (Figs. 2I, K, 3C). The body diameter of swimming specimens typically ranges from 55–65 μm, and 45.7–54.7 μm after dry silver nitrate impregnation. The macronucleus is C- or horseshoe-shaped, centrally located, approximately 55 μm in diameter, 10 μm wide, and 140 μm long in the “uncoiled” condition after protargol impregnation. It is clearly visible in vivo due to its brightness in contrast to the green symbiotic algae filling the cytoplasm. Nucleoli are roughly globular to ellipsoidal, small to medium-sized, and densely and evenly distributed over the macronucleus, well recognizable after protargol impregnation (Figs. 2C, I, 3C). The micronucleus was not observed in vivo or after protargol impregnation. A single contractile vacuole, approximately 9 μm across during diastole, is located centrally (Fig. 2F, J). The ciliate appears pale green due to the presence of numerous globular green algae (zoochlorellae), each 3–5 μm in diameter. The cytoplasm is colorless, containing numerous ~ 0.5 μm-sized granules and several brownish food vacuoles measuring 7–15 μm (Fig. 2E–I).

Description and measurements of the attachment apparatus are based on silver nitrate-impregnated specimens (Fig. 3A, B; Table 1). The adhesive disc measures 41.3–48.6 μm in diameter and is surrounded by a border membrane 1.9–3.1 μm wide. The denticle ring is 26.2–30.8 μm in diameter; the exring measures 36.7–43.9 μm. The ring comprises 26–30 denticles, each with 9–11 radial pins. Denticle span is 8.7–13.1 μm; denticle length is 5.7–7.8 μm. The ratio of denticle length above and below the *x* axis is 0.9–1.4. The blade is well-developed, sickle-shaped with a rounded distal margin, 4.6–5.8 μm long and 2.3–3.2 μm wide, occupying about two-thirds of the space between the *y* and *y* + 1 axes. The blade apophysis is inconspicuous; the anterior margin slightly extends beyond the *y* + 1 axis, and the posterior margin forms a shallow arch relative to the *y* axis (Figs. 2A, 3A). The tangent point is distinct and located at or below the level of the distal blade surface (Fig. 2A). The central part is well defined, 1.0–2.7 μm wide; the minimum width of the section connecting to the blade is 0.4–0.9 μm and to the ray 0.4–1.2 μm. No posterior projection is present (Figs. 2A, 3A). Rays are robust, extending parallel to the *y* axes, 3.1–6.2 μm long, with a generally smooth outline, only rarely irregular, and tapering gradually to acute tips. Anterior apophysis of the ray is absent. No granules are present in the center of the adhesive disc (Figs. 2A, H, J, 3A).

The aboral ciliary wreath encircles the outer perimeter of the adhesive disc and is conspicuous in vivo due to its densely arranged cilia, each 12–15 μm long. It consists of three rings (inner, middle, and outer) of narrowly spaced basal bodies, clearly visible after protargol impregnation (Figs. 2B, 3D, E). The inner ring comprises two adjacent circles of dikinetids bearing peripheral pins. The middle ring consists of four circles of dikinetids forming transversely oriented polykinetids with locomotory cilia, positioned distinctly closer to the outer than to the inner ring. The outer ring is composed of a single circle of dikinetids bearing marginal cilia.

The oral apparatus is inconspicuous in vivo and appears as a furrow containing the adoral ciliary spiral, visible at 1000× magnification (Fig. 2K). The adoral ciliary spiral performs ~ 1.13 turns (407°) around the peristomial disc and is composed of a haplokinety running alongside a polykinety (Figs. 2C, 3F, H, I). The haplokinety originates at the base of the infundibulum and consists of very densely arranged, obliquely oriented dikinetids. The polykinety is composed of numerous short, oblique rows of basal bodies, each row containing three basal bodies. Upon entering the infundibulum, the polykinety differentiates into three buccal membranelles (peniculi 1–3), whose fine structure corresponds to that of the polykinety (Figs. 2C, D, 3F, G, J, K). Peniculus 1 is a direct continuation of the external polykinety, extending in a sigmoidal pattern to the base of the infundibulum. Peniculus 2 originates where basal body rows are gradually added from left to right at the transition point between the polykinety and peniculus 1. It runs parallel to peniculus 1 and appears to intersect it optically near the proximal end of the infundibulum. Peniculus 3 is distinctly shorter than peniculi 1 and 2. It begins near their termini at the base of the infundibulum and extends in a C-shaped pattern toward the proximal end of the haplokinety. The germinative kinety is composed of obliquely oriented dikinetids. It extends along the haplokinety from the distal three-fourths of the infundibulum and terminates as a tail of widely spaced dikinetids after exiting the infundibulum (Figs. 2C, D, 3F, G, J, K).

### Host, prevalence, and intensity

During our investigation of trichodinids associated with freshwater gastropods, *T. chlorophora* was detected exclusively in *Ph. acuta* collected from the Vojčianske jazero (lake). Nearly all examined snails were infected, each harboring approximately 20‒30 ciliates. No trichodinids were found in the co-occurring gastropod *Lymnaea stagnalis* (Linneus, 1758).

To date, *T. chlorophora* has been reported only from North America. It was originally described from *Ph. ampullacea* (now considered a junior synonym of *Ph. gyrina*) and *Ph. traskii* (I. Lea, 1864) [*species inquirenda* according to MolluscaBase eds. 2025], collected from ponds near Stanford University, southern California (Richards 1948). In addition, *T. chlorophora* was recorded in *Ph. gyrina* from ponds and streams in Jackson County, Illinois (Blecka and Garoian 1972). According to Richards (pers. comm. in Blecka and Garoian 1972), the species was also found in various physiid snails across a broad range, from Maryland to Florida and Washington to California. Blecka and Garoian (1972) reported that in a sample of 150 *P. gyrina* specimens, all were infected with 20 to 50 ciliates per snail, and all ciliates contained numerous zoochlorellae.

### Characterization of new sequences

From 13 Slovak specimens of *T. chlorophora*, a total of 52 new sequences were obtained from the macronuclear 18S rRNA gene and the ITS1-5.8S-ITS2-28S rRNA region as well as from the mitochondrial 16S rRNA and COI genes. These sequences have been deposited in GenBank (https://www.ncbi.nlm.nih.gov/genbank/) under the following accession numbers: PV770921–PV770933, PV770895–PV770907, PV770908–PV770920, and PV769974–PV769986, respectively.

The 18S rRNA gene of *T. chlorophora* was 1701 nucleotides long and had a GC content of 49.85%. No intraspecific variability was detected. According to a BLASTN search, the closest relative is *T. unionis* (GenBank accession KY596041), with a sequence identity of 99.44%, followed by the three species of the *T. steinii* group: *T. steinii*, *T. polycelis* (both 97.36%), and *T. schmidtea* (97.41%). Species-specific nucleotide differences distinguishing *T. chlorophora* from *T. unionis* (KY596041) were located in helix 16 of the 5′ domain (four mutations) and helix 41 of the 3′M domain (one mutation). Representatives of the *T. steinii* group also differed from *T. chlorophora* in helices 16 (five mutations) and 41 (five mutations), and additionally in helix 21 within the hypervariable V4 region of the C domain (Supplementary Fig. S1).

The amplified ITS1-5.8S-ITS2-28S rRNA gene region of *T. chlorophora* was 921 nucleotides long, with a GC content of 51.14%. The ITS1 region measured 92 nucleotides, the 5.8S rRNA gene 139 nucleotides, the ITS2 region 79 nucleotides, and the D1 domain of the 28S rRNA gene 611 nucleotides, including 12 nucleotides at the 3’ end that belong to the D0 domain (Supplementary Fig. S2). As with the 18S rRNA gene, no intraspecific variation was observed. Since no ITS-28S region sequences are available for *T. unionis* (KY596041), members of the *T. steinii* group were the top matches in the BLASTN search. However, *T. chlorophora* differed from *T. steinii* by 8.53‒8.88%, from *T. polycelis* by 8.53‒8.65%, and from *T. schmidtea* by 8.75%. The predicted secondary structures of the 5.8S rRNA, the first barcoding domain of the 28S rRNA, and the ITS2 molecule were consistent with those of the *T. steinii* group. Most unique nucleotide characters were located in the hypervariable helix 25 and its expansion regions (Supplementary Fig. S2).

**Fig. 4:**
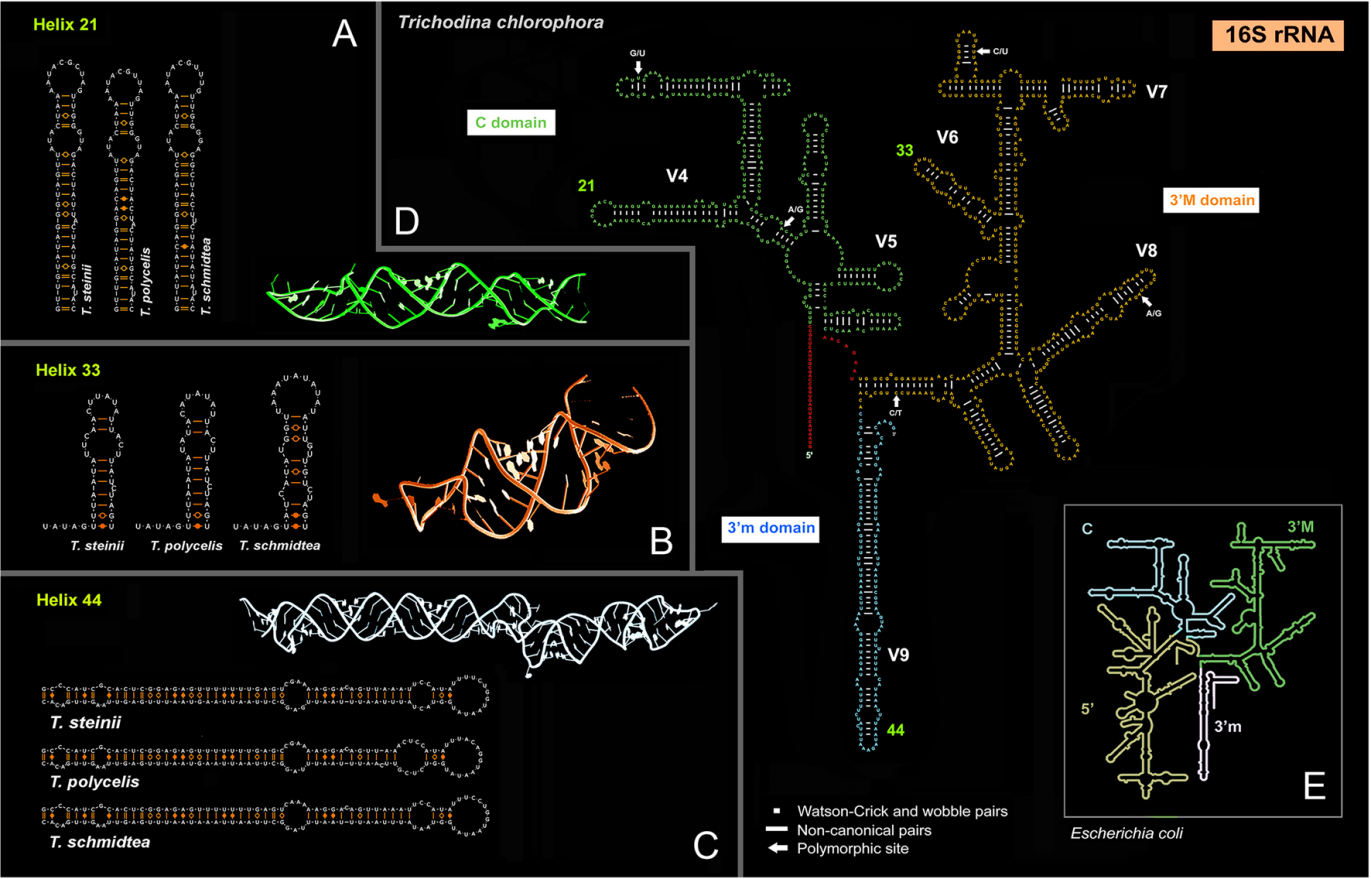
Secondary structure model of the mitochondrial 16S rRNA molecule of *Trichodina chlorophora* (**D**) and comparison with closely related taxa (**A–C**). The secondary structure prediction is based on the *Escherichia coli* model, considering the 3D ribosomal structure. **E** The secondary structure map of the *E. coli* 16S rRNA molecule (lower right panel) is from http://apollo.chemistry.gatech.edu/RibosomeGallery (Petrov et al. 2014). Helices from representatives of the *T. steinii* group are from Zhang et al. (2023). Three-dimensional models of helices 21, 33, and 44 were predicted using RNAComposer (Popenda et al. 2012)

The amplified region of the mitochondrial 16S rRNA gene spanned the C, 3′M, and 3′m domains. It was consistently 920 nucleotide long and had a GC content of 30.87‒31.30%. Among the 13 *T. chlorophora* sequences analyzed, five polymorphic sites were identified (indicated by arrows in Fig. 4), corresponding to an intraspecific divergence of up to 0.6%. As with the ITS-28S region, representatives of the *T. steinii* group were the top matches in the BLASTN searches. *Trichodina chlorophora* differed from *T. steinii* by 15.15‒15.89%, from *T. polycelis* by 15.03‒15.46%, and from *T. schmidtea* by 15.48‒15.80%. Moreover, *T. chlorophora* could be distinguished from the members of the *T. steinii* group by the structure of helix 21 in the hypervariable V4 region of the C domain, helix 33 in the 3′M domain, and helix 44 in the 3′m domain (Fig. 4).

The amplified mitochondrial COI gene fragment of *T. chlorophora* was consistently 765 nucleotides in length, had a GC content of 25.62‒25.75%, and exhibited only two variable nucleotide positions, corresponding to an intraspecific divergence of up to 0.3%. The top matches in the BLASTX search were again members of the *T. steinii* group, which differed from *T. chlorophora* by as much as 34.4‒35.5% in nucleotide sequence.

### Phylogenetic affinity of members of the hyper-symbiotic system

The host heterobranch snail was DNA barcoded as *Ph. acuta* using the mitochondrial COI gene. It shared 96.53‒99.85% sequence identity with other members of the *Ph. acuta* complex and was most similar (99.85% identity) to specimens collected from both North America and Europe.

**Fig. 5:**
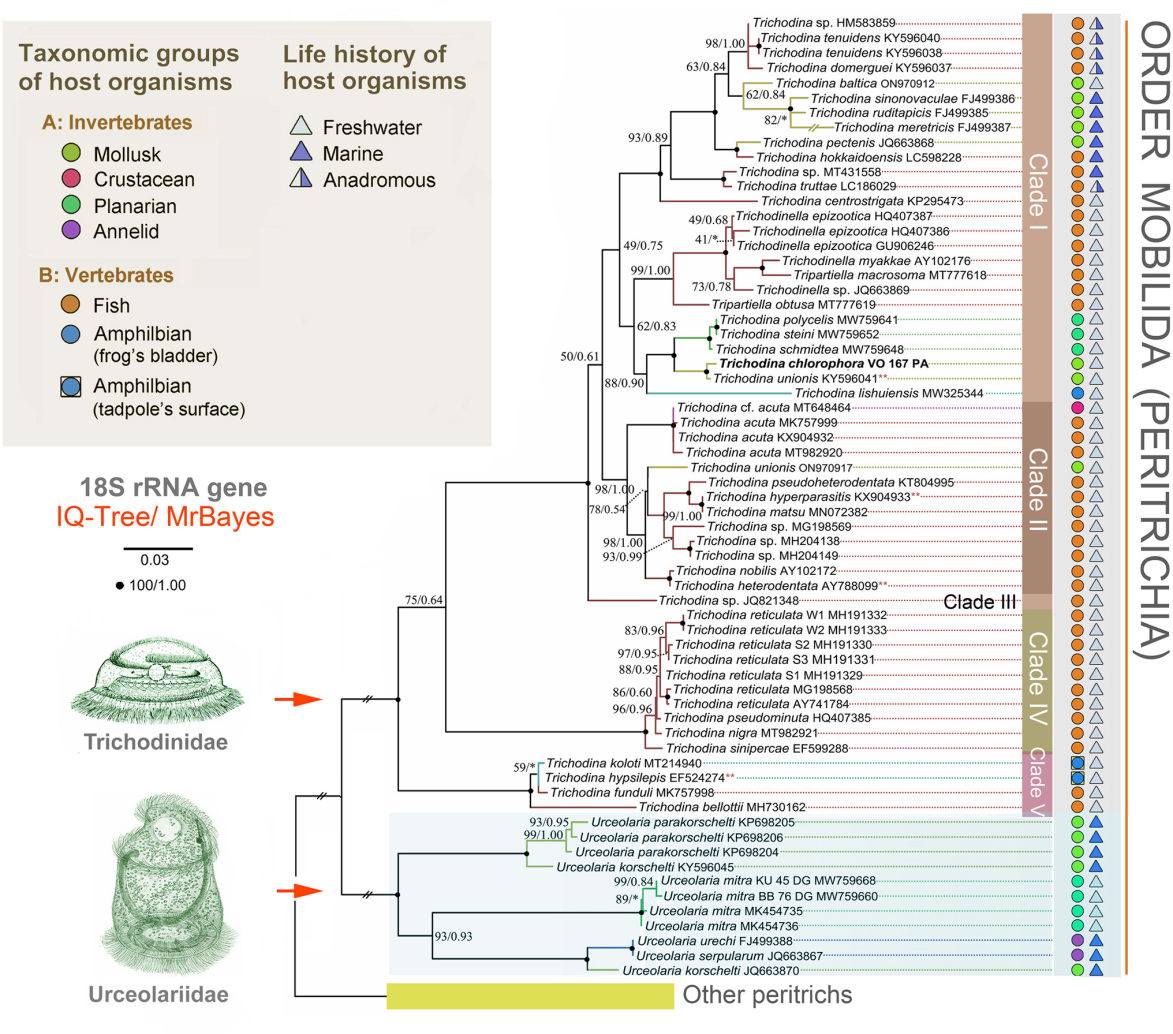
Phylogenetic tree based on nuclear 18S rRNA gene sequences, showing the systematic positions of *Trichodina chlorophora*. Bootstrap values from maximum likelihood analysis (IQ-TREE) and posterior probabilities from Bayesian inference (MrBayes) are mapped onto the best-scoring IQ-TREE topology. Fully statistically supported nodes (bootstrap = 100%, posterior probability = 1.00) are marked with solid black circles. An asterisk (*) indicates topological incongruence between the two methods; double-asterisks (**) denote synonymous names or suspected misidentifications (see Supplementary Table S1 for details). The scale bar represents three nucleotide substitutions per 100 positions

**Fig. 6:**
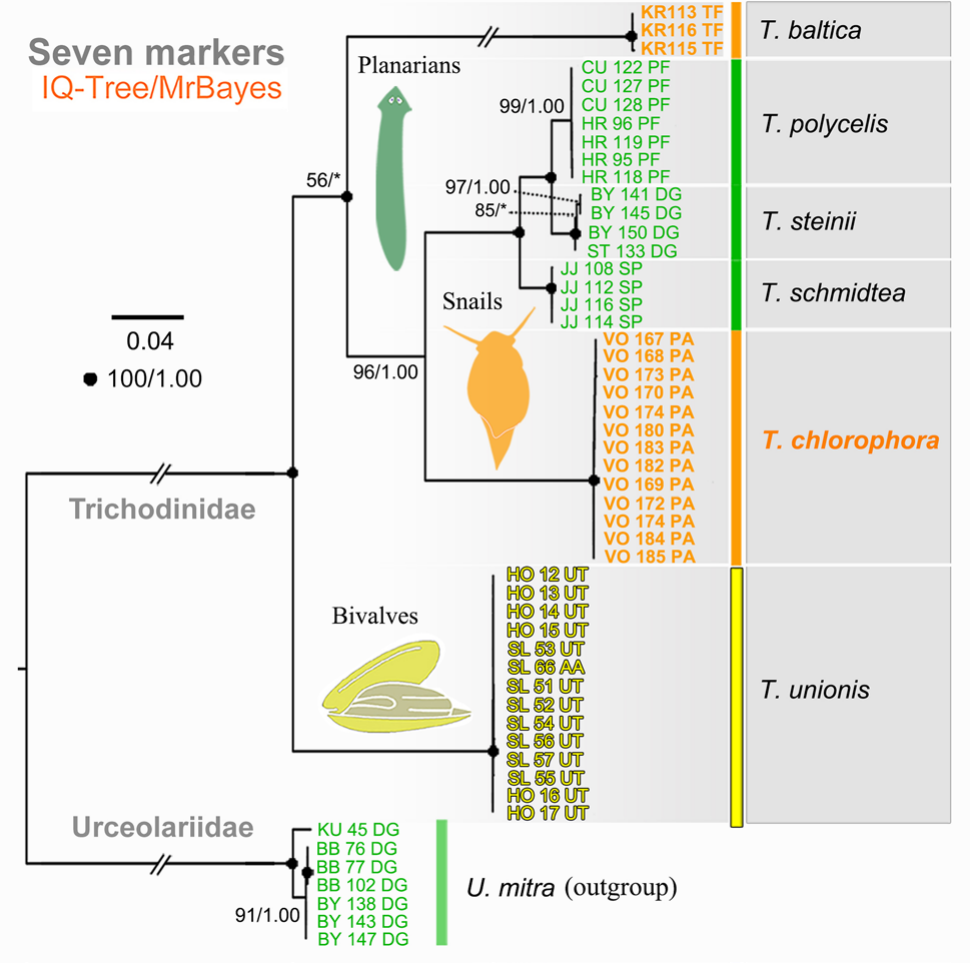
Phylogenetic tree based on two mitochondrial (16S and COI) and five nuclear (18S, ITS1, 5.8S, ITS2, and D1-28S) markers, showing the systematic positions of *Trichodina chlorophora*. Bootstrap values from maximum likelihood analysis (IQ-TREE) and posterior probabilities from Bayesian inference (MrBayes) are mapped onto the best-scoring IQ-TREE topology. Fully statistically supported nodes (bootstrap = 100%, posterior probability = 1.00) are marked with solid black circles. An asterisk (*) indicates topological incongruence between the two methods. The scale bar represents four nucleotide substitutions per 100 positions

In 18S phylogenies, *T. chlorophora* was nested within the freshwater trichodinid clade comprising an endobiont of frogs and epibionts of mollusks and planarians (88% ML bootstrap/0.90 posterior probability). It clustered with full support alongside *T. unionis* (KY596041), which was isolated from the freshwater snail *Stagnicola* sp. in North America. This sequence did not group with the ‘true’ *T. unionis*, which is associated with river mussels of the genera *Unio* and *Anodonta* native to Europe. The *T. chlorophora* + *T. unionis* (KY596041) clade was sister to the *T. steinii* group, which includes three planarian-dwelling species reported from Europe (Fig. 5). This branching pattern was also recovered in phylogenetic trees inferred from two mitochondrial (16S and COI) and five nuclear (18S, ITS1, 5.8S, ITS2, and D1-28S) markers (Fig. 6). Due to the absence of ITS-28S, 16S, and COI sequence data for *T. unionis* (KY596041), *T. chlorophora* grouped with strong support with the *T. steinii* group in the multigene tree (96% ML bootstrap/1.00 posterior probability).

**Fig. 7:**
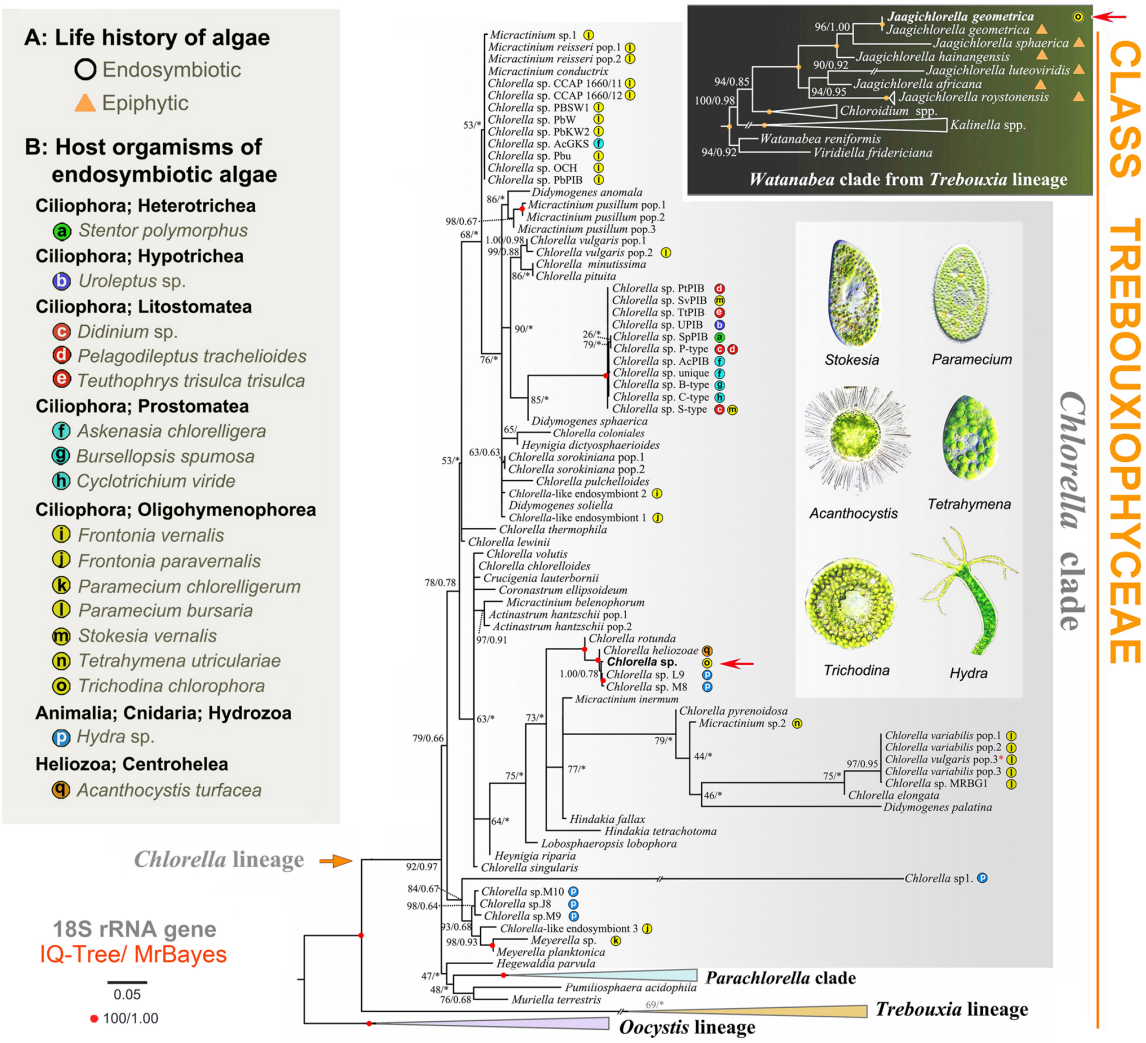
Phylogenetic tree based on nuclear 18S rRNA gene sequences, showing the systematic positions of green algae associated with *Trichodina chlorophora* (marked with red arrows). Bootstrap support values from maximum likelihood analysis (IQ-TREE) and posterior probabilities from Bayesian inference (MrBayes) are shown on the best-scoring IQ-TREE topology. Nodes with full statistical support (bootstrap = 100%, posterior probability = 1.00) are marked with solid red circles. Asterisks (*) denote topological incongruence between the two methods. The scale bar represents three nucleotide substitutions per one hundred positions. The image of *Acanthocystis* was sourced from the website realmicrolife.com, which permits image use for scientific purposes. The remaining five images were provided by the authors and their collaborators

**Fig. 8:**
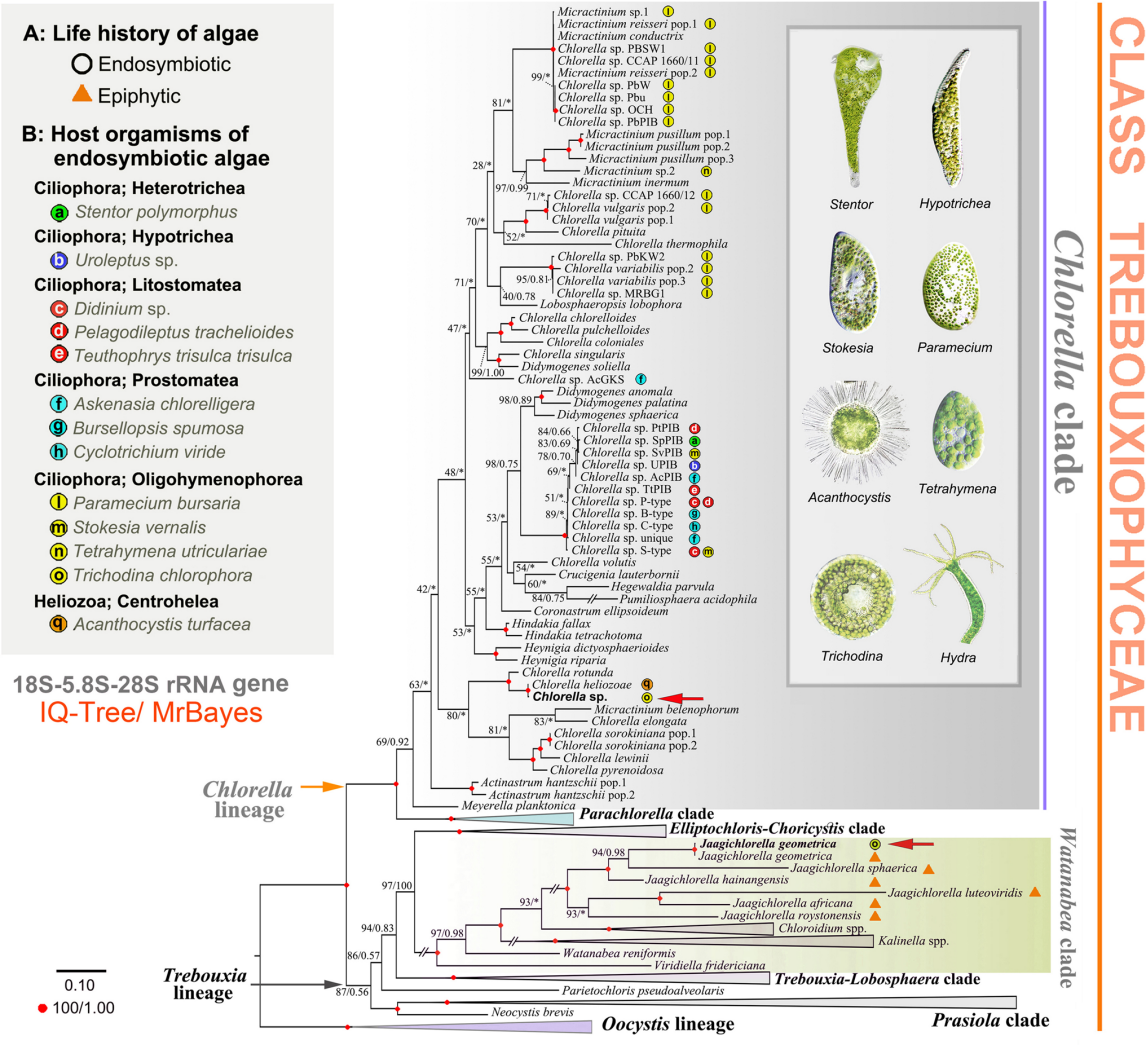
Phylogenetic tree based on concatenated nuclear 18S, 5.8S, and 28S rRNA gene sequences, showing the systematic positions of green algae associated with *Trichodina chlorophora* (marked with red arrows). Bootstrap support values from maximum likelihood analysis (IQ-TREE) and posterior probabilities from Bayesian inference (MrBayes) are shown on the best-scoring IQ-TREE topology. Nodes with full statistical support (bootstrap = 100%, posterior probability = 1.00) are marked with solid red circles. Asterisks (*) indicate topological incongruence between the two methods. The scale bar represents three nucleotide substitutions per one hundred positions. Images of *Acanthocystis*, *Stentor*, *Paramecium*, and *Teuthophrys* were sourced from the websites realmicrolife.com and ciliates.at.com, both of which permit use for scientific purposes. The remaining images, including those of the hypotrich, *Hydra* and *Trichodina*, were provided by the authors and their collaborators

All *T. chlorophora* specimens contained numerous pale green zoochlorellae. DNA barcoding using 18S and ITS1-5.8S-ITS2-28S sequences consistently revealed the presence of two algal species: *Chlorella* sp. TchVO and *Jaagichlorella geometrica* (Figs. 7, 8). *Chlorella* sp. TchVO formed a fully supported clade with the free-living *Ch. rotunda*, *Ch. heliozoae* (from *Acanthocystis turfacea*, Haptista: Centrohelea), and *Chlorella* sp. L9 and M8 (from the *Hydra viridissima* group, Cnidaria). *Jaagichlorella geometrica*, obtained from *T. chlorophora*, was identical to the previously reported epiphytic strain *J. geometrica* SAG2549 (GenBank accession MH780944). It clustered with other congeners in a fully supported subclade within the *Watanabea* clade of the *Trebouxia* lineage (Figs. 7, 8). Our phylogenetic analyses of the class Trebouxiophyceae indicate that the tendency toward endosymbiosis is widespread in the *Chlorella* clade but rare in the *Watanabea* clade. Moreover, taxa within the *Chlorella* clade do not cluster according to host organisms, suggesting low phylogenetic host specificity.

## Discussion

Integrating detailed morphological descriptions with molecular taxonomy is crucial for elucidating the evolution and biogeography of symbiotic systems, particularly in ciliates, where true diversity is often cryptic. Accordingly, we conducted a comprehensive morpho-molecular characterization of *T. chlorophora* and evaluated the identity of the Slovak population in comparison with data from North American populations (Blecka and Garoian 1972; Richards 1948). By examining the phylogenetic affiliations of all three partners in this hyper-symbiotic system, we tested three hypotheses: (1) trichodinids colonized aquatic snails multiple times independently; (2) trichodinids associated with aquatic snails have a broad geographic range and host spectrum; and (3) endosymbiotic green algae exhibit low phylogenetic host specificity.

### Identity of the Slovak population of *Trichodina chlorophora*

*Trichodina chlorophora* was originally described by Richards (1948) from the mantle cavity of physinine snails in southern California. It was later reported across the continental United States (Blecka and Garoian 1972), but until now, it had not been recorded outside North America. In this study, we identified *T. chlorophora* in an invasive physinine snail of North American origin (*Physella acuta*), as confirmed by a 99.85% sequence identity in the COI gene with North American specimens (Lydeard et al. 2016; Young et al. 2021).

Richards (1948) and Blecka and Garoian (1972) provided sufficient biometric data to confidently identify the Slovak population as *T. chlorophora* (see Table 2). Specifically, the body diameter, adhesive disc diameter, denticle ring width, ray length, and the number of denticles and radial pins per denticle in Slovak specimens all fall within the ranges reported for the type population. Only minor differences (less than 1 μm) were observed in the width of the border membrane and the central part of the denticle (Table 2). Combined with the presence of zoochlorellae and the association with physinine snails, these findings strongly support the conspecificity of the Slovak population with *T. chlorophora*.

**Table 2:** Biometric comparison of *Trichodina chlorophora* populations

Character/reference	Present study	Richards (1948)	Blecka and Garoian (1972)
Body, diameter	50.2 (45.7‒54.7)	55.5 (41‒70)	65.7 (48‒83)
Adhesive disc, diameter	45.2 (41.3‒48.6)	46.4 (37‒59)	44.7 (36‒65)
Border membrane, width	2.5 (1.9‒3.1)	4.1	2.2 (1.5‒3.0)
Denticle ring, diameter	28.2 (26.2‒30.8)	30.1 (23‒38)	27.8 (24‒39)
Denticle, number	27.5 (26.0‒30.0)	27 (23‒30)	27 (24‒29)
Denticle, length	6.5 (5.7‒7.8)	‒	7.25
Ray, length	4.8 (3.1‒6.2)	4.4 (2.0‒5.5)	5.0 (4.0‒7.0)
Central part, width	1.6 (1.0‒2.7)	1.9 (1.6‒2.5)	1.6 (1.0‒2.0)
Blade, length	5.2 (4.6‒5.8)	‒	5.0 (4.0‒7.0)
Radial pins per denticle, number	10.1 (9.0‒11.0)	9	9‒10
Zoochlorellae	Present	Present	Present
Geographic location	Vojčianske jazero (lake), Slovakia	Ponds near Stanford University, southern California, U.S.A	Ponds and streams in Jackson County, Illinois, U.S.A
Host	*Physella acuta*	*Physella ampullacea*^a^	*Physella gyrina*

All measurements are in μm

^a^Now considered a junior synonym of *Physella gyrina* (Say, 1821) (MolluscaBase eds. 2025)

*Trichodina chlorophora* can be distinguished from three other congeners reported from physinine snails (*T. breviradiosa* Richards, 1948; *T. physellarum* Richards, 1948; and *T. helisomarum* Richards, 1948) based on features of the adhesive apparatus and the presence or absence of zoochlorellae. The ray in *T. chlorophora* is significantly longer (3.1–7.0 μm, mean 4.7 μm) than in *T. breviradiosa* (1.5–3.0 μm, mean 2.4 μm), but shorter than in *T. helisomarum* (8.0–12.0 μm, mean 9.1 μm). Furthermore, both *T. helisomarum* and *T. physellarum* can be readily distinguished from *T. chlorophora* by the absence of zoochlorellae.

### Independent colonization of aquatic snails by trichodinids

Zhang et al. (2023) proposed that the progenitor of the genus *Trichodina* colonized freshwater fishes before the breakup of Gondwana, explaining the genus’ global distribution across all continents except Antarctica. Their analyses suggest that *Trichodina* radiated following the second major phase of Pangaea’s breakup (150‒140 Ma), with at least four independent colonization events of mollusks from poikilothermic vertebrate hosts. Our phylogenetic analyses support this scenario: snail-dwelling taxa did not form a monophyletic group (Fig. 5), corroborating our first hypothesis.

The evolutionary history of *Trichodina* appears to be shaped by host switching from vertebrates to invertebrates, followed by diversification within newly colonized lineages. No reverse transfers from invertebrates back to vertebrates were detected. However, host shifts among unrelated invertebrates are common and often lead to speciation and stable associations with the new host or its close relatives (Rataj and Vďačný 2021; Zhang et al. 2023). This pattern is also consistently supported by the present phylogenetic analyses (Figs. 5, 6).

### Geographic range and host spectrum of snail-dwelling trichodinids

Blecka and Garoian (1972) suggested that trichodinids associated with aquatic snails have broad geographic ranges and host spectra, consistent with the perceived ubiquity and host promiscuity of symbiotic ciliates. Our findings partially support this view. The European population of *T. chlorophora* morphologically matches North American populations described from physinine snails (Blecka and Garoian 1972; Richards 1948; Table 2), indicating at least a Holarctic distribution. However, we isolated *T. chlorophora* exclusively from *Ph. acuta*, a North American species (Lydeard et al. 2016; Young et al. 2021), and never from any native European or Asian gastropods or bivalves, even when they co-occurred with *Ph. acuta* (Li et al. 2024; Zhang and Vďačný 2021, 2023a, 2024; Zhang et al. 2023; present study). This suggests that the broad distribution of *T. chlorophora* is likely a result of human-mediated dispersal of its invasive host, which was introduced into southern Europe probably with ornamental aquatic plants in the eighteenth century and reached Central Europe by the mid-twentieth century (Horsák et al. 2013). Similar anthropogenic range expansions have been documented for other symbiotic ciliates, such as tetrahymenids associated with invasive gastropods (Zhang and Vďačný 2023b).

Although structural host specificity in snail-dwelling trichodinids appears low (several species have been reported from multiple gastropods), their phylogenetic host specificity is high. For instance, *T. chlorophora*, *T. breviradiosa*, *T. physellarum*, and *T. helisomarum* have been found exclusively in physinine snails (Blecka and Garoian 1972; Richards 1948; present study), *T. baltica* only in nerites (for a review, see Zhang et al. 2023), and *T. tranquillis* and *T. planorbicola* only in planorbids (Machado Filho 1965; Raabe and Raabe 1961). Apparent broad host spectra in earlier studies may reflect misidentifications, as demonstrated for the bivalve-associated *T. unionis* by Zhang et al. (2023) using molecular tools.

Lynn (2008) proposed that the distribution of symbiotic ciliates mirrors that of their hosts. If snail-dwelling trichodinids exhibit high phylogenetic host specificity and their hosts have distinct biogeographies, then trichodinid distributions should reflect those of their hosts. However, human-mediated host dispersal and subsequent host switching in new environments must also be considered (Zhang and Vďačný 2023b). Our findings, therefore, do not fully support the second hypothesis: although *T. chlorophora* exhibits a broad geographic distribution, this pattern is more likely attributable to anthropogenic dispersal than to intrinsic ecological generalism.

### Low phylogenetic host specificity of endosymbiotic green algae

Endosymbiotic green algae are commonly found in a variety of ciliates, heliozoans (e.g., *Acanthocystis*), and invertebrates such as *Hydra* and *Spongilla*. These algae have traditionally been referred to as *Chlorella* and *Chlorella*-like algae, or collectively as zoochlorellae (Pröschold et al. 2011). Phylogenetic analyses have shown that these endosymbionts are polyphyletic, originating from multiple lineages within the classes Trebouxiophyceae and Chlorophyceae (e.g., Hoshina et al. 2005, 2021; Kawaida et al. 2013; Kreutz et al. 2012; Muñoz-Gómez et al. 2021; Pitsch et al. 2017; Pröschold et al. 2011). Endosymbionts of ciliates alone derive from at least ten independent Trebouxiophyceae lineages, particularly within the genera *Chlorella*, *Meyerella*, and *Micractinium* (Figs. 7, 8).

These algae do not cluster according to their host organisms (Figs. 7, 8), indicating low phylogenetic host specificity. This is further supported by experimental studies showing that aposymbiotic ciliates can establish stable symbioses with *Chlorella*-like strains isolated from unrelated hosts such as other ciliates or *Hydra*, but not with free-living *Chlorella* species (Summerer et al. 2007). Summerer et al. (2008) also demonstrated that endosymbionts from six different ciliate species collected from the same pond were nearly identical in their 18S and ITS1 sequences, regardless of host species. Conversely, endosymbionts from the same ciliate species but different ponds were genetically distinct. Based on these findings, Pröschold et al. (2011) proposed that ciliates form stable symbioses with green algae that are locally available and susceptible to endosymbiosis.

In the Slovak population of *T. chlorophora*, we detected the simultaneous presence of rDNA and ITS sequences from two green algal species. One of them, *Chlorella* sp. TchVO, formed a fully supported clade with *Ch. heliozoae* (isolated from the heliozoan *Acanthocystis turfacea*) and with *Chlorella* sp. L9 and M8 (associated with the *Hydra viridissima* group). The second alga was molecularly identified as *J. geometrica*. Neither species clustered with endosymbiotic *Chlorella* and *Chlorella*-like strains previously isolated from ciliates (Figs. 7, 8). These findings support the hypothesis proposed by Pröschold et al. (2011), which suggests that any susceptible green alga may potentially establish an endosymbiotic partnership with a suitable ciliate host.

Our study is the first to report a representative of *Jaagichlorella* from a zoochlorellae-bearing ciliate. Most *Jaagichlorella* species are known as photobionts of lichens or as epiphytes on rocks and artificial substrates (Darienko and Pröschold 2019). Therefore, we cannot exclude the possibility that the *J. geometrica* sequences originated from prey algae growing epiphytically on the snail shell. However, it has been demonstrated that *Paramecium bursaria* can form stable symbioses with at least four green algal species: *Scenedesmus* (Chlorophyceae) and *Coccomyxa simplex*, *Ch. vulgaris*, and *Ch. fusca* var. *vacuolata* (Trebouxiophyceae) (Pröschold et al. 2011).

Although our phylogenetic analyses support the third hypothesis, several questions remain. Was *T. chlorophora* introduced to Europe along with its North American endosymbiotic green algae? Has it also formed stable symbioses with green algae native to European habitats? Do symbionts of American and European origin coexist in European *T. chlorophora* populations, or have the European symbionts replaced the original ones? To answer these questions, molecular analyses of zoochlorellae from the type population of *T. chlorophora* in ponds near Stanford University are needed.

## Conclusion

The following conclusions can be drawn from this study:

1. Independent colonization of aquatic snails by trichodinids. Trichodinid ciliates have colonized aquatic snails multiple times independently from poikilothermic vertebrate hosts, as evidenced by their scattered phylogenetic positions and host-switching patterns.
2. High phylogenetic host specificity in trichodinids. Although some trichodinids appear to have broad host ranges, molecular data reveal high phylogenetic host specificity. Their current geographic distributions may have been, however, artificially expanded through human-mediated transmission of their host organisms.
3. Low host specificity in green algal endosymbionts. Endosymbiotic green algae exhibit low phylogenetic and structural host specificity. Any susceptible green alga may potentially form a stable endosymbiotic relationship with any compatible ciliate, depending on local availability and ecological conditions.
4. Convergent evolution of tripartite symbioses. The parallel emergence of green algae-bearing trichodinids in both physinine and planorbid snails suggests convergent evolution. This may reflect co-evolutionary processes that independently gave rise to complex symbiotic associations among aquatic snails, ciliates, and zoochlorellae.

## Supplementary Information

Below is the link to the electronic supplementary material.

Supplementary file1 (PDF 1253 KB)

## Data Availability

All data generated or analyzed during this study are included in this published article and supplementary material and can be found in online repositories. The names of the repositories and accession numbers can be found at: https://www.ncbi.nlm.nih.gov/genbank/.
