# Supplementary material for: Morpho-molecular characterization of Trichodina chlorophora Richards, 1948 (Protista: Ciliophora), a central component in the ‘snail‒ciliate‒zoochlorellae’ hyper-symbiotic system
Source: Mar Life Sci Technol. 2026 Apr 7;8(2):371–86. doi: 10.1007/s42995-026-00359-4 (PMC13198607; doi:10.1007/s42995-026-00359-4)
Supplement: Supplementary file 1 — Supplementary file1 (PDF 1253 KB) [file 42995_2026_359_MOESM1_ESM.pdf]

SUPPLEMENTARY MATERIAL

Morpho-molecular characterization of *Trichodina chlorophora* Richards, 1948 (Protista, Ciliophora), a central component in the 'snail–ciliate–zoochlorellae' hyper-symbiotic system

Tengyue Zhang[1,2] · Peter Vďačný[2]

[1] The Key Laboratory of Zoological Systematics and Application, College of Life Sciences, Hebei University, Baoding 071002, China

[2] Department of Zoology, Faculty of Natural Sciences, Comenius University in Bratislava, 842 15 Bratislava, Slovak Republic

✉ Peter Vďačný
peter.vdacny@uniba.sk

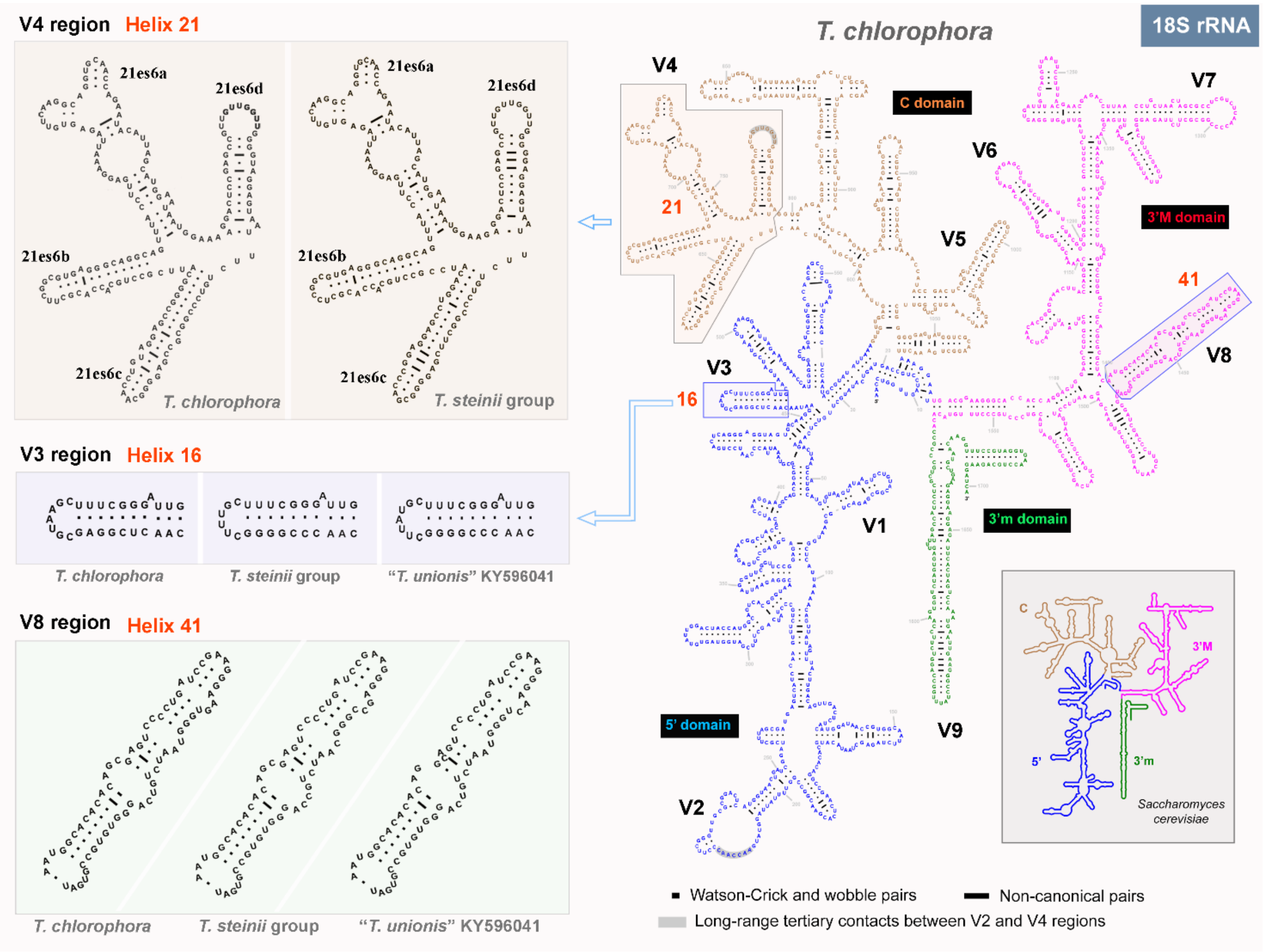

Suppl. Fig. S1 Secondary structure model of the macronuclear 18S rRNA molecule of *Trichodina chlorophora* and comparison with closely related taxa. The secondary structure prediction is based on the *Saccharomyces cerevisiae* model, considering the 3D ribosomal structure. The secondary structure map of the *S. cerevisiae* 18S rRNA molecule (lower right panel) is from http://apollo.chemistry.gatech.edu/RibosomeGallery (Petrov et al. 2014)

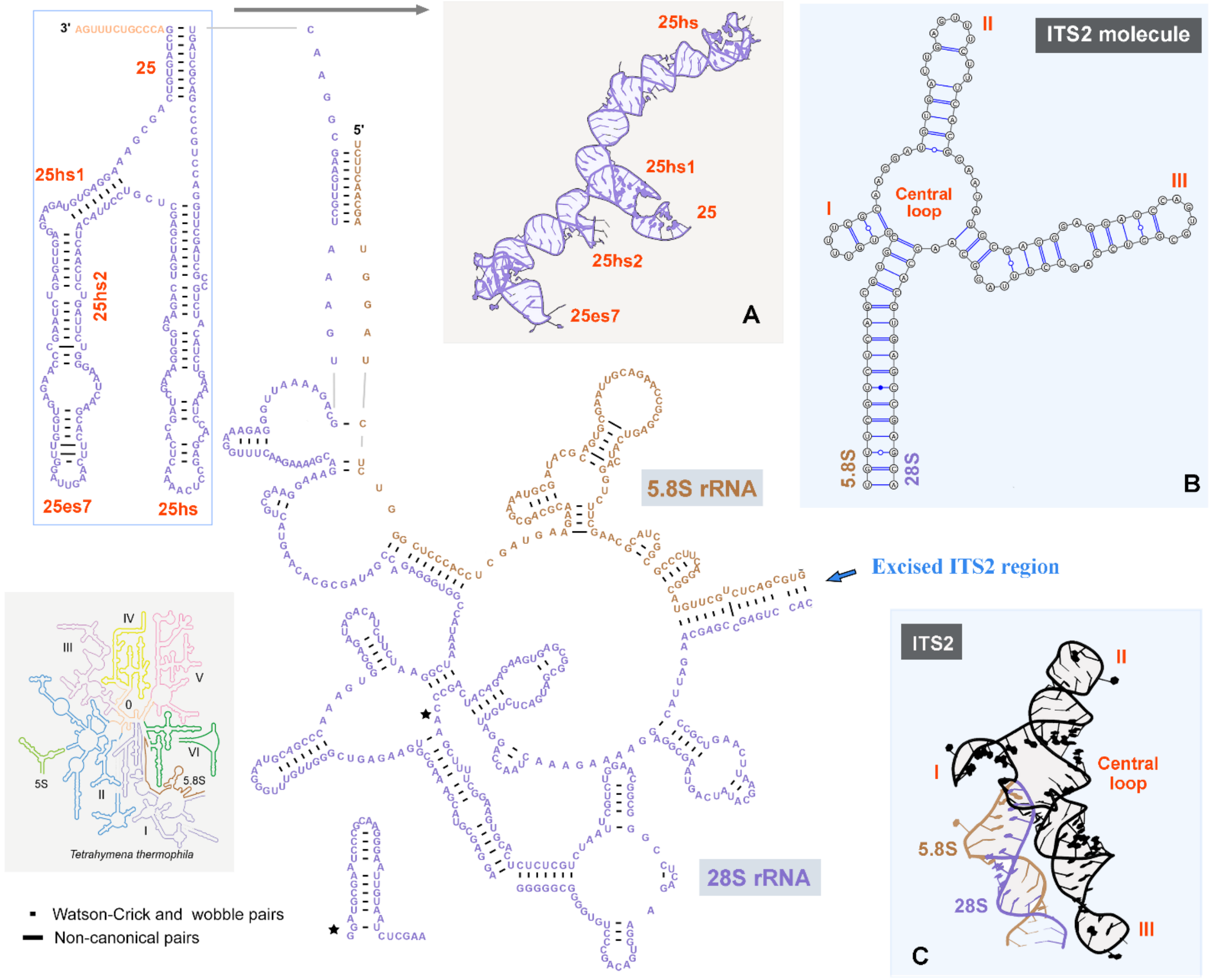

Suppl. Fig. S2 Secondary structure models of the macronuclear 5.8S rRNA, the first domain of the 28S rRNA, and the ITS2 molecule of *Trichodina chlorophora*. The secondary structure prediction of the 5.8S-28S rRNA molecules is based on the *Tetrahymena thermophila* model, considering the 3D ribosomal structure. The secondary structure map of the *T. thermophila* 5.8S and 28S rRNA molecules (lower left panel) is from http://apollo.chemistry.gatech.edu/RibosomeGallery (Petrov et al. 2014). **A** Three-dimensional model of the hypervariable helix 25. **B, C** Two- and three-dimensional models of the ITS2 molecule, including the imperfect hybridization helix 10 formed by 3′-end of 5.8S and 5′-end of 28S. The three helices radiating from the central loop of the ITS2 molecule are labeled with Roman numerals I–III

Supplementary Table S1

Origin and GenBank accession numbers of mobilid 18S rRNA gene sequences used to construct the 18S rRNA gene trees

Taxon	GenBank accession number	Host organism	Higher taxonomic group of host	Life history of host	Reference
Trichodina acuta pop. 1	KX904932	*Misgurnus anguillicaudatus*	Fish	Freshwater	Wang et al. (2017)
Trichodina acuta pop. 2	MT982920	*Cyprinus carpio*	Fish	Freshwater	Wang et al. (2022)
Trichodina acuta pop. 3	MK757999	*Cyprinus carpio*	Fish	Freshwater	Wang et al. (2020)
Trichodina acuta TAJF01	MT648464	*Notodiaptomus* sp.	Copepod	Freshwater	de Oliveira Furtado et al. (2020)
Trichodina baltica KR 112 TF	ON970912	*Theodoxus flutviatilis*	Mollusk	Freshwater	Zhang et al. (2023)
Trichodina bellottii	MH730162	*Austrolebias bellottii*	Fish	Freshwater	Marcotegui et al. (2018)
Trichodina centrostrigata	KP295473	*Oreochromis aureus*	Fish	Freshwater	Wang et al. (2015)
Trichodina chlorophora VO 167 PA	PV770921	*Physella acuta*	Mollusk	Freshwater	Present study
Trichodina domerguei	KY596037	*Gasterosteus aculeatus*	Fish	Anadromous	Irwin et al. (2017)
Trichodina funduli	MK757998	*Paramisgurnus dabryanus*	Fish	Freshwater	Wang et al. (2020)
Trichodina heterodentata[a]	AY788099	*Ctenopharyngodon idella*	Fish	Freshwater	Gong et al. (2006)
Trichodina hokkaidoensis	LC598228	*Verasper moseri*	Fish	Marine	Mizuno et al. (2021)
Trichodina hyperparasitis[b]	KX904933	*Pelteobagrus fulvidraco*	Fish	Freshwater	Wang et al. (2017)
Trichodina hypsilepis[c]	EF524274	*Rana* sp.	Amphibian	Freshwater	Gong (2007)
Trichodina koloti	MT214940	*Sclerophrys gutteralis*	Amphibian	Freshwater	de Jager et al. (2019)
Trichodina lishuiensis	MW325344	*Odorrana schmackeri*	Amphibian	Freshwater	Zhou et al. (2021b)
Trichodina matsu	MN072382	*Tachysurus fulvidraco*	Fish	Freshwater	Zhou et al. (2021a)
Trichodina meretricis	FJ499387	*Meretrix lyreta*	Mollusk	Marine	Zhan et al. (2009)
Trichodina nigra	MT982921	*Cyprinus carpio*	Fish	Freshwater	Wang et al. (2022)
Trichodina nobilis	AY102172	*Hypophthalmichtys molitrix*	Fish	Freshwater	Gong (2007)
Trichodina sp.	MG198569	*Micropercops swinhonis*	Fish	Freshwater	Wang et al. (2018)
Trichodina pectenis	JQ663868	*Patinopecten yessoensis*	Mollusk	Marine	Zhan et al. (2013)
T. polycelis HR 95 PF	MW759641	*Polycelis felina*	Planarian	Freshwater	Rataj and Vďačný (2021)
T. pseudoheterodentata	KT804995	*Ictalurus punctatus*	Fish	Freshwater	Tang et al. (2017)
Trichodina pseudominuta	HQ407385	*Carassius auratus*	Fish	Freshwater	Tang and Zhao (2016)

Taxon	GenBank accession number	Host organism	Higher taxonomic group of host	Life history of host	Reference
Trichodina reticulata pop. 1	AY741784	*Ctenopharyngodon idella*	Fish	Freshwater	Gong et al. (2006)
Trichodina reticulata pop. 2	MG198568	*Micropercops swinhonis*	Fish	Freshwater	Wang et al. (2018)
Trichodina reticulata S1	MH191329	*Carassius auratus*	Fish	Freshwater	Wang et al. (2019)
Trichodina reticulata S2	MH191330	*Carassius auratus*	Fish	Freshwater	Wang et al. (2019)
Trichodina reticulata S3	MH191331	*Carassius auratus*	Fish	Freshwater	Wang et al. (2019)
Trichodina reticulata W1	MH191332	*Ctenopharyngodon idella*	Fish	Freshwater	Wang et al. (2019)
Trichodina reticulata W2	MH191333	*Ctenopharyngodon idella*	Fish	Freshwater	Wang et al. (2019)
Trichodina ruditapicis	FJ499385	*Ruditapes philippinarum*	Mollusk	Marine	Zhan et al. (2009)
Trichodina schmidtea JJ 108 SP	MW759648	*Schmidtea polychroa*	Planarian	Freshwater	Rataj and Vďačný (2021)
Trichodina sinipercae	EF599288	*Ctenopharyngodon idella*	Fish	Freshwater	Gong (2007)
Trichodina sinonovaculae	FJ499386	*Sinonovacula constricta*	Mollusk	Marine	Zhan et al. (2009)
Trichodina sp. B4-D22	MH204149	*Channa argus*	Fish	Freshwater	Tang et al. (unpublished)
Trichodina sp. CCo-2020	MT431558	*Lycodes* spp.	Fish	Marine	Collins et al. (unpublished)
Trichodina sp. FS	HM583859	*Salmo salar*	Fish	Anadromous	Nylund and Isaksen (unpublished)
Trichodina sp. TEL-2012	JQ821348	*Clarias gariepinus*	Fish	Freshwater	Isaksen et al. (unpublished)
Trichodina sp. Ydu-2019	MH204138	*Channa argus*	Fish	Freshwater	Du et al. (unpublished)
Trichodinella sp. ZZ-2012	JQ663869	*Ctenopharyngodon idella*	Fish	Freshwater	Zhan et al. (2013)
Trichodina steinii BB 104 DG	MW759652	*Dugesia gonocephala*	Planarian	Freshwater	Rataj and Vďačný (2021)
Trichodina tenuidens HL-1	KY596038	*Gasterosteus aculeatus*	Fish	Anadromous	Irwin et al. (2017)
Trichodina tenuidens UBC-6	KY596040	*Gasterosteus aculeatus*	Fish	Anadromous	Irwin et al. (2017)
Trichodina truttae	LC186029	*Oncorhynchus keta*, *O. masou*, *O. mykiss*, *Salvelinus leucomaenis*, *S. malma*	Fish	Anadromous	Mizuno et al. (2016)
Trichodina unionis HO 12 UT	ON970917	*Unio tumidus*	Mollusk	Freshwater	Zhang et al. (2023)
Trichodina unionis 29_BB1[d]	MN082436	*Gyraulus siamensis*, *Physella acuta*	Mollusk	Freshwater	Wiroonpan and Purivirojkul (2019)
Trichodina unionis 99_BB1[d]	MN082437	*Gyraulus siamensis*, *Physella acuta*	Mollusk	Freshwater	Wiroonpan and Purivirojkul (2019)
Trichodina unionis UBC-2[d]	KY596041	*Stagnicola* sp.	Mollusk	Freshwater	Irwin et al. (2017)
Trichodinella epizootica (C)	HQ407386	*Carassius auratus*	Fish	Freshwater	Zhang et al. (2015)
Trichodinella epizootica (D)	HQ407387	*Cyprinus carpio*	Fish	Freshwater	Zhang et al. (2015)

Taxon	GenBank accession number	Host organism	Higher taxonomic group of host	Life history of host	Reference
Trichodinella epizootica (H)	GU906246	*Carassius auratus*	Fish	Freshwater	Zhang et al. (2015)
Trichodinella myakkae	AY102176	*Ctenopharyngodon idella*	Fish	Freshwater	Gong et al. (2006)
Tripartiella macrosoma	MT777618	*Tachysurus fulvidraco*	Fish	Freshwater	Qin et al. (2021)
Tripartiella obtusa	MT777619	*Hemibarbus maculatus*	Fish	Freshwater	Qin et al. (2021)
Urceolaria korschelti	JQ663870	*Solen strictus*	Mollusk	Marine	Zhan et al. (2013)
Urceolaria korschelti C1	KY596045	*Lepidochitona cinerea*	Mollusk	Marine	Irwin et al. (2017)
Urceolaria mitra BB 76 DG	MW759660	*Dugesia gonocephala*	Planarian	Freshwater	Rataj and Vďačný (2021)
Urceolaria mitra C15/8	MK454735	*Dugesia gonocephala*	Planarian	Freshwater	Rataj and Vďačný (2019)
Urceolaria mitra KU 45 DG	MW759668	*Dugesia gonocephala*	Planarian	Freshwater	Rataj and Vďačný (2021)
Urceolaria mitra RT45/5	MK454736	*Dugesia gonocephala*	Planarian	Freshwater	Rataj and Vďačný (2019)
Urceolaria parakorschelti L1	KP698205	*Lottia pelta*	Mollusk	Marine	Irwin et al. (2017)
Urceolaria parakorschelti L42	KP698204	*Lottia pelta*	Mollusk	Marine	Irwin et al. (2017)
Urceolaria parakorschelti L47	KP698206	*Lottia pelta*	Mollusk	Marine	Irwin et al. (2017)
Urceolaria serpularum	JQ663867	*Serpula* sp.	Annelid	Marine	Zhan et al. (2013)
Urceolaria urechi	FJ499388	*Urechis unicinctus*	Annelid	Marine	Zhan et al. (2009)

[a] *Trichodina heterodentata* (AY788099) is considered to be a synonym of *T. hypsilepis* by de Jager et al. (2019)

[b] *Trichodina hyperparasitis* (KX904933) is considered to be a misidentified *T. matsu* (MN072382) according to Zhou et al. (2021b)

[c] *T. hypsilepis* (EF524274) is considered to be a synonym of *T. koloti* by de Jager et al. (2019)

[d] *Trichodina unionis* 29_BB1 (MN082436), *T. unionis* 99_BB1 (MN082437), and *T. unionis* UBC-2 (KY596041) are misidentifications according to Zhang et al. (2023) and they very likely represent new species

References

de Jager GP, Basson L, van Marwijk J (2019) A new *Trichodina* species (Peritrichia: Mobilida) from anuran tadpole hosts, *Sclerophrys* spp. in the Okavango Panhandle, Botswana, with comments on this taxon. Acta Protozool 58:141–153. https://doi.org/10.4467/16890027AP.19.014.11915

de Oliveira Furtado EJ, Cedrola F, Senra MVX, de Oliveira Marchesini R, Romero-Niembro V, Dias RJP (2020) Morphology, molecular phylogeny, and taxonomy of trichodinids (Ciliophora, Mobilida) from Calanoid copepods. Parasitol Res 119:2597–2608. https://doi.org/10.1007/s00436-020-06741-0

Gong Y (2007) Studies on the phylogeny of trichodinids. Dissertation, Institute of Hydrobiology, Chinese Academy of Sciences

Gong Y, Yu Y, Villalobo E, Zhu F, Miao W (2006) Reevaluation of the phylogenetic relationship between mobilid and sessilid peritrichs (Ciliophora, Oligohymenophorea) based on small subunit rRNA genes sequences. J Eukaryot Microbiol 53:397–403. https://doi.org/10.1111/j.1550-7408.2006.00121.x

Irwin NAT, Sabetrasekh M, Lynn DH (2017) Diversification and phylogenetics of mobilid peritrichs (Ciliophora) with description of *Urceolaria parakorschelti* sp. nov. Protist 168:481–493. https://doi.org/10.1016/j.protis.2017.07.003

Marcotegui PS, Montes MM, Barneche J, Ferrari W, Martorelli S (2018) Geometric morphometric on a new species of Trichodinidae. A tool to discriminate trichodinid species combined with traditional morphology and molecular analysis. Int J Parasitol: Parasites Wildl 7:228–236. https://doi.org/10.1016/j.ijppaw.2018.06.004

Mizuno S, Matsuda T, Nishikawa S, Ito S (2021) Morphological and molecular phylogenetic analyses of an ectoparasitic *Trichodinid* ciliate, *Trichodina hokkaidoensis* n. sp., infecting artificially reared barfin flounder *Verasper moseri.* Fish Pathol 56:115–121. https://doi.org/10.3147/jsfp.56.115

Mizuno S, Urawa S, Miyamoto M, Hatakeyama M, Saneyoshi H, Sasaki Y, Koide N, Ueda H (2016) The epidemiology of the trichodinid ciliate *Trichodina truttae* on hatchery-reared and wild salmonid fish in Hokkaido. Fish Pathol 51:199–209. https://doi.org/10.3147/jsfp.51.199

Qin W, Zhou Q, Wang S, Tang F, Zhao Y (2021) Molecular characterization and phylogenetic analyses of *Tripartiella macrosoma* Basson and Van As, 1987 and *Tripartiella obtusa* Ergens and Lom, 1970 based on 18S rRNA gene sequence data. Parasitol Res 120:2391–2399. https://doi.org/10.1007/s00436-021-07136-5

Rataj M, Vďačný P (2019) Living morphology and molecular phylogeny of oligohymenophorean ciliates associated with freshwater turbellarians. *Dis Aquat Org* 134:147–166. https://doi.org/10.3354/dao03366

Rataj M, Vďačný P (2021) Cryptic host-driven speciation of mobilid ciliates epibiotic on freshwater planarians. Mol Phylogenet Evol 161:e107174. https://doi.org/10.1016/j.ympev.2021.107174

Tang F, Zhao Y (2016) Molecular phylogenetic evidences on Mobilida based on genetic distance and GC content of 18S rDNA using broad taxon sampling. Acta Hydrobiol Sin 40:358–369.

Tang F, Zhang Y, Zhao Y (2017) Morphological and molecular identification of the new species, *Trichodina pseudoheterodentata* sp. n. (Ciliophora, Mobilida, Trichodinidae) from the channel catfish, *Ictalurus punctatus*, in Chongqing China. J Eukaryot Microbiol 64:45–55. https://doi.org/10.1111/jeu.12335

Wang Q, Tang F, Zhao Y (2015) Clone and sequence analysis of 18S rDNA of *Trichodina centrostrigata* Basson, Van As & Paperna, 1983. J Chongqing Norm Univ (Nat Sci Ed) 32:4 (in Chinese). https://doi.org/10.11721/cqnuj20150421

Wang S, Zhao Y, Du Y, Tang F (2019) Morphological redescription and molecular identification of *Trichodina reticulata* Hirschmann & Partsch, 1955 (Ciliophora, Mobilida, Trichodinidae) with the supplemental new data of SSU rDNA and ITS-5.8S rDNA. J Eukaryot Microbiol 66:447–459. https://doi.org/10.1111/jeu.12689

Wang Z, Bourland WA, Zhou T, Yang H, Zhang C, Gu Z (2020) Morphological and molecular characterization of two *Trichodina* (Ciliophora, Peritrichia) species from freshwater fishes in China. Eur J Protistol 72:e125647. https://doi.org/10.1016/j.ejop.2019.125647

Wang Z, Deng Q, Zhou T, Yang H, Gu Z (2018) First record of two ectoparasitic ciliates of the genus *Trichodina* (Ciliophora: Trichodinidae) parasitizing gills of an invasive freshwater fish, *Micropercops swinhonis*, in Tibet. Parasitol Res 117:2233–2242. https://doi.org/10.1007/s00436-018-5910-y

Wang Z, Liu M, Ma H, Lu B, Shen Z, Mu C, Alfarraj SA, El-Serehy HA, Warren A (2022) Redescription and molecular characterization of two *Trichodina* species (Ciliophora, Peritrichia, Mobilida) from freshwater fish in China. Parasitol Int 86:e102470. https://doi.org/10.1016/j.parint.2021.102470

Wang Z, Zhou T, Gu Z (2017) New data of two trichodinid ectoparasites (Ciliophora: Trichodinidae) from farmed freshwater fishes in Hubei, China. Eur J Protistol 60:50–59. https://doi.org/10.1016/j.ejop.2017.04.002

Wiroonpan P, Purivirojkul W (2019) New record of *Trichodina unionis* (Ciliophora, Trichodinidae) from freshwater gastropods in Bangkok, Thailand. Parasite 26:e47. https://doi.org/10.1051/parasite/2019047

Zhan Z, Xu K, Dunthorn M (2013) Evaluating molecular support for and against the monophyly of the Peritrichia and phylogenetic relationships within the Mobilida (Ciliophora, Oligohymenophorea). Zool Scr 42:213–226. https://doi.org/10.1111/j.1463-6409.2012.00568.x

Zhan Z, Xu K, Warren A, Gong Y (2009) Reconsideration of phylogenetic relationships of the subclass Peritrichia (Ciliophora, Oligohymenophorea) based on small subunit ribosomal RNA gene sequences, with the establishment of a new subclass Mobilia Kahl, 1933. J Eukaryot Microbiol 56:552–558. https://doi.org/10.1111/j.1550-7408.2009.00435.x

Zhang T, Rurik I, Vďačný P (2023) A holistic approach to inventory the diversity of mobilid ciliates (Protista: Ciliophora: Peritrichia). Org Divers Evol 23:425–454. https://doi.org/10.1007/s13127-022-00601-8

Zhang Y, Zhao Y, Wang Q, Tang F (2015) New comparative analysis based on the secondary structure of SSU-rRNA gene reveals the evolutionary trend and the family-genus characters of Mobilida (Ciliophora, Peritrichia). Curr Microbiol 71:259–267. https://doi.org/10.1007/s00284-015-0848-0

Zhou Q, Tang F, Zhao Y (2021a) Morphological redescription and molecular characterization of *Trichodina matsu* Basson & Van As, 1994 (Ciliophora, Mobilida, Trichodinidae) infecting *Tachysurus fulvidraco* (Richardson, 1846) from Chongqing, China. Zootaxa 4995:334–344. https://doi.org/10.11646/zootaxa.4995.2.6

Zhou W, Wang R, Zhao W, Zou H, Li W, Wu S, Li M, Wang G (2021b) A new species of *Trichodina lishuiensis* n. sp. (Ciliophora: Trichodinidae) in urinary bladder of *Odorrana schmackeri* (Amphibia: Ranidae) from Zhejiang, China. Acta Trop 221:e106015. https://doi.org/10.1016/j.actatropica.2021.106015

Supplementary Table S2

Origin and GenBank accession numbers of mobilid sequences used to construct the multigene trees

Species	Specimen	Host organism	GenBank accession number			
			18S	ITS-28S	16S	COI
T. chlorophora	VO 167 PA	*Physella acuta*	PV770921	PV770895	PV770908	PV769974
	VO 168 PA	*Physella acuta*	PV770922	PV770896	PV770909	PV769975
	VO 169 PA	*Physella acuta*	PV770923	PV770897	PV770910	PV769976
	VO 170 PA	*Physella acuta*	PV770924	PV770898	PV770911	PV769977
	VO 171 PA	*Physella acuta*	PV770925	PV770899	PV770912	PV769978
	VO 172 PA	*Physella acuta*	PV770926	PV770900	PV770913	PV769979
	VO 173 PA	*Physella acuta*	PV770927	PV770901	PV770914	PV769980
	VO 174 PA	*Physella acuta*	PV770928	PV770902	PV770915	PV769981
	VO 180 PA	*Physella acuta*	PV770929	PV770903	PV770916	PV769982
	VO 182 PA	*Physella acuta*	PV770930	PV770904	PV770917	PV769983
	VO 183 PA	*Physella acuta*	PV770931	PV770905	PV770918	PV769984
	VO 184 PA	*Physella acuta*	PV770932	PV770906	PV770919	PV769985
	VO 185 PA	*Physella acuta*	PV770933	PV770907	PV770920	PV769986
T. baltica	KR 113 TF	*Theodoxus fluviatilis*	ON970913	ON985383	ON970933	ON968465
	KR 115 TF	*Theodoxus fluviatilis*	ON970915	ON985385	ON970935	ON968466
	KR 116 TF	*Theodoxus fluviatilis*	ON970916	ON985386	ON970936	ON968467
T. polycelis	HR 95 PF	*Polycelis felina*	MW759641	MW759669	MW768981	MW759776
	HR 96 PF	*Polycelis felina*	MW759642	MW759670	MW768982	MW759777
	HR 118 PF	*Polycelis felina*	MW759643	MW759671	MW768983	MW759778
	HR 119 PF	*Polycelis felina*	MW759644	MW759672	MW768984	MW759779
	CU 122 PF	*Polycelis felina*	MW759645	MW759673	MW768985	MW759780
	CU 127 PF	*Polycelis felina*	MW759646	MW759674	MW768986	MW759781
	CU 128 PF	*Polycelis felina*	MW759647	MW759675	MW768987	MW759782
T. schmidtea	JJ 108 SP	*Schmidtea polychroa*	MW759648	MW759676	MW768988	MW759783
	JJ 112 SP	*Schmidtea polychroa*	MW759649	MW759677	MW768989	MW759784
	JJ 114 SP	*Schmidtea polychroa*	MW759650	MW759678	MW768990	MW759785
	JJ 116 SP	Schmidtea polychroa	MW759651	MW759679	MW768991	MW759786
T. steinii	BY 141 DG	*Dugesia gonocephala*	MW759655	MW759683	MW768995	MW759787
	BY 145 DG	*Dugesia gonocephala*	MW759656	MW759684	MW768996	MW759788
	BY 150 DG	*Dugesia gonocephala*	MW759657	MW759685	MW768997	MW759789
	ST 133 DG	*Dugesia gonocephala*	MW759658	MW759686	MW768998	MW759790
T. unionis	HO 12 UT	*Unio tumidus*	ON970917	ON985387	ON970937	ON968468
	HO 13 UT	*Unio tumidus*	ON970918	ON985388	ON970938	ON968469
	HO 14 UT	*Unio tumidus*	ON970919	ON985389	ON970939	ON968470
	HO 15 UT	*Unio tumidus*	ON970920	ON985390	ON970940	ON968471

Species	Specimen	Host organism	GenBank accession number			
			**18S**	**ITS-28S**	**16S**	**COI**
	HO 16 UT	*Unio tumidus*	ON970921	ON985391	ON970941	ON968472
	HO 17 UT	*Unio tumidus*	ON970922	ON985392	ON970942	ON968473
	SL 51 UT	*Unio tumidus*	ON970923	ON985393	ON970943	ON968474
	SL 52 UT	*Unio tumidus*	ON970924	ON985394	ON970944	ON968475
	SL 53 UT	*Unio tumidus*	ON970925	ON985395	ON970945	ON968476
	SL 54 UT	*Unio tumidus*	ON970926	ON985396	ON970946	ON968477
	SL 55 UT	*Unio tumidus*	ON970927	ON985397	ON970947	ON968478
	SL 56 UT	*Unio tumidus*	ON970928	ON985398	ON970948	ON968479
	SL 57 UT	*Unio tumidus*	ON970929	ON985399	ON970949	ON968480
	SL 66 AA	*Anodonta anatina*	ON970930	ON985400	ON970950	ON968481
U. mitra	BB 76 DG	*Dugesia gonocephala*	MW759660	MW759688	MW768900	MW759791
	BB 77 DG	*Dugesia gonocephala*	MW759661	MW759689	MW768901	MW759792
	BB 102 DG	*Dugesia gonocephala*	MW759664	MW759692	MW768902	MW759795
	BY 138 DG	*Dugesia gonocephala*	MW759665	MW759693	MW768903	MW759796
	BY 143 DG	*Dugesia gonocephala*	MW759666	MW759694	MW768904	MW759797
	BY 147 DG	*Dugesia gonocephala*	MW759667	MW759695	MW768905	MW759798
	KU 45 DG	*Dugesia gonocephala*	MW759668	MW759697	MW768907	MW759799

Supplementary Table S3

Origin and GenBank accession numbers of green algae sequences used to construct the 18S rRNA gene and multigene trees

Taxon	Symbiotic or epiphytic strain	Host organism	Higher taxonomic group of host	GenBank accession number		Reference
				18S	ITS-5.8S-28S	
Actinastrum hantzschii pop.1	no	–	–	FM205884	FM205884	Luo et al. (2010)
Actinastrum hantzschii pop.2	no	–	–	AF288365	FM205841	Luo et al. (2010)
Asterochloris magna UTEX 902	yes	*Pilophorus aciculare*	Lichenized Ascomycota	KP318691	–	Škaloud et al. (2015)
Chlorella chlorelloides	no	–	–	HQ111432	HQ111432	Pitsch et al. (2017)
Chlorella coloniales	no	–	–	FM205862	FM205862	Pitsch et al. (2017)
Chlorella elongata	no	–	–	FM205858	FM205858	Pitsch et al. (2017)
Chlorella heliozoae	yes	*Acanthocystis turfacea*	Heliozoa; Centrohelea	FM205850	FM205850	Pitsch et al. (2017)
Chlorella lewinii	no	–	–	FM205861	FM205861	Pitsch et al. (2017)
Chlorella minutissima	no	–	–	FM205861	FM205861	Pitsch et al. (2017)
Chlorella pituita	no	–	–	EF030564	–	Summerer et al. (2008)
Chlorella pulchelloides	no	–	–	HQ111430	HQ111430	Pitsch et al. (2017)
Chlorella pyrenoidosa	no	–	–	AB240151	LR215766	Serra et al. (2021)
Chlorella rotunda	no	–	–	HQ111433	HQ111433	Pitsch et al. (2017)
Chlorella singularis	no	–	–	HQ111435	HQ111435	Pitsch et al. (2017)
Chlorella sorokiniana pop.1	no	–	–	FM205859	FM205859	Luo et al. (2010)
Chlorella sorokiniana pop.2	no	–	–	FM205860	FM205860	Luo et al. (2010)
Chlorella sp. AcGKS	yes	*Askenasia chlorelligera* GKS	Ciliophora; Prostomatea	EF030560	EF030577	Summerer et al. (2008)
Chlorella sp. AcPIB	yes	*Askenasia chlorelligera* PIB	Ciliophora; Prostomatea	EF030559	EF030576	Summerer et al. (2008)
Chlorella sp. B-type	yes	*Bursellopsis spumosa*	Ciliophora; Prostomatea	LC075788	LC075788	Hoshina and Kusuoka (2016)
Chlorella sp. CCAP 1660/11	yes	*Paramecium bursaria*	Ciliophora; Oligohymenophorea	AB206548	AB206548	Summerer et al. (2008)
Chlorella sp. CCAP 1660/12	yes	*Paramecium bursaria*	Ciliophora; Oligohymenophorea	AB260894	AB260895	Hoshina and Imamura (2008)
Chlorella sp. C-type	yes	*Cyclotrichium viride*	Ciliophora; Prostomatea	LC075792	LC075792	Hoshina and Kusuoka (2016)
Chlorella sp. L9	yes	*Hydra* sp. L9	Animalia; Cnidaria	AB713412	–	Kawaida et al. (2013)
Chlorella sp. M8	yes	*Hydra* sp. M9	Animalia; Cnidaria	AB713413	–	Kawaida et al. (2013)
Chlorella sp. MRBG1	yes	*Paramecium bursaria*	Ciliophora; Oligohymenophorea	AB219527	AB219527	Summerer et al. (2008)
Chlorella sp. OCH	yes	*Paramecium bursaria*	Ciliophora; Oligohymenophorea	EF030561	EF030578	Summerer et al. (2008)

Taxon	Symbiotic or epiphytic strain	Host organism	Higher taxonomic group of host	GenBank accession number		Reference
				18S	ITS-5.8S-28S	
Chlorella sp. PbKM2	yes	*Paramecium bursaria*	Ciliophora; Oligohymenophorea	EF030567	EF030584	Summerer et al. (2008)
Chlorella sp. PbPIB	yes	*Paramecium bursaria*	Ciliophora; Oligohymenophorea	EF030565	EF030582	Summerer et al. (2008)
Chlorella sp. PBSW1	yes	*Paramecium bursaria*	Ciliophora; Oligohymenophorea	AB206547	AB206547	Summerer et al. (2008)
Chlorella sp. Pbu	yes	*Paramecium bursaria*	Ciliophora; Oligohymenophorea	EF030562	EF030579	Summerer et al. (2008)
Chlorella sp. PbW	yes	*Paramecium bursaria*	Ciliophora; Oligohymenophorea	EF030566	EF030583	Summerer et al. (2008)
Chlorella sp. PtPIB	yes	*Pelagodileptus trachelioides*	Ciliophora; Litostomatea	EF030556	EF030573	Summerer et al. (2008)
Chlorella sp. P-type	yes	*Pelagodileptus trachelioides*/ *Didinium* sp.	Ciliophora; Litostomatea	LC075784	LC075784	Hoshina and Kusuoka (2016)
Chlorella sp. SpPIB	yes	*Stentor polymorphus*	Ciliophora; Heterotrichea	EF030558	EF030575	Summerer et al. (2008)
Chlorella sp. S-type	yes	*Stokesia vernalis*/*Didinium* sp.	Ciliophora; Oligohymenophorea/ Litostomatea	LC075779	LC075779	Hoshina and Kusuoka (2016)
Chlorella sp. SvPIB	yes	*Stokesia vernalis*	Ciliophora; Oligohymenophorea	EF030555	EF030572	Summerer et al. (2008)
Chlorella sp. TtPIB	yes	*Teuthophrys trisulca trisulca*	Ciliophora; Litostomatea	EF030557	EF030574	Summerer et al. (2008)
Chlorella sp. unique	yes	*Didinium* sp.	Ciliophora; Litostomatea	LC075798	LC075798	Hoshina and Kusuoka (2016)
Chlorella sp. UPIB	yes	*Uroleptus* sp.	Ciliophora; Hypotrichea	EF030554	EF030571	Summerer et al. (2008)
Chlorella sp. J8	yes	*Hydra* sp. J8	Animalia; Cnidaria	AB713408	–	Kawaida et al. (2013)
Chlorella sp. M10	yes	*Hydra* sp. strain M10	Animalia; Cnidaria	AB713410	–	Kawaida et al. (2013)
Chlorella sp. M9	yes	*Hydra* sp. M9	Animalia; Cnidaria	AB713409	–	Kawaida et al. (2013)
Chlorella thermophila	no	–	–	KF661334	KJ002639	Serra et al. (2021)
Chlorella variabilis pop.1	yes	*Paramecium bursaria*	Ciliophora; Oligohymenophorea	AB260893	AB260893	Hoshina and Imamura (2008)
Chlorella variabilis pop.3	yes	*Paramecium bursaria*	Ciliophora; Oligohymenophorea	FM205849	FM205849	Luo et al. (2010)
*Chlorella variabilis*pop.2	yes	*Paramecium bursaria*	Ciliophora; Oligohymenophorea	AB206550	ON167513	Summerer et al. (2008)
Chlorella volutis	no	–	–	HQ111434	HQ111434	Serra et al. (2021)
Chlorella vulgaris pop.1	unknown	–	–	FM205854	FM205854	Luo et al. (2010)
Chlorella vulgaris pop.2	yes	*Paramecium bursaria*	Ciliophora; Oligohymenophorea	AB260895	AB260895	Hoshina and Imamura (2008)
Chlorella vulgaris pop.3[a]	yes	*Paramecium bursaria*	Ciliophora; Oligohymenophorea	AB191207	–	Hoshina et al. (2004)
Chlorella sp1.	yes	*Hydra viridis*	Animalia; Cnidaria	AB206551	AB288025	Hoshina and Imamura (2008)
Chlorella sp. TchVO	yes	*Trichodina chlorophora*	Ciliophora; Oligohymenophorea	PX870620	PX870620	Present study
Chlorella-like endosymbiont 1	yes	*Frontonia vernalis*	Ciliophora; Oligohymenophorea	MT040853	–	Serra et al. (2021)
Chlorella-like endosymbiont 2	yes	*Frontonia paravernalis*	Ciliophora; Oligohymenophorea	MT040852	–	Serra et al. (2021)

Taxon	Symbiotic or epiphytic strain	Host organism	Higher taxonomic group of host	GenBank accession number		Reference
				18S	ITS-5.8S-28S	
Chlorella-like endosymbiont 3	yes	*Frontonia paravernalis*	Ciliophora; Oligohymenophorea	MT040851	–	Serra et al. (2021)
Chloroidium ellipsoideum	no	–	–	FM946015	FM946015	Pitsch et al. (2017)
Chloroidium engadiense	no	–	–	FM946011	FM946011	Darienko and Pröschold (2019)
Chloroidium laureanum	no	–	–	MH551522	MH551522	Darienko and Pröschold (2019)
Chloroidium lobatum	no	–	–	MH551523	MH551523	Darienko and Pröschold (2019)
Chloroidium saccharophilum	no	–	–	FM946000	FM946000	Darienko and Pröschold (2019)
Choricystis parasitica	no	–	–	FN298929	FN298929	Darienko et al. (2019)
Closteriopsis acicularis	no	–	–	FM205847	FM205847	Luo et al. (2010)
Coccomyxa sp. CCAP 1660/13	yes	*Paramecium bursaria*	Ciliophora; Oligohymenophorea	AB260896	AB260896	Hoshina and Imamura (2008)
Coccomyxa subellipsoidea	no	–	–	HG972978	HG972978	Darienko et al. (2019)
Compactochlorella kochii	no	–	–	HQ322124	HQ322124	Pitsch et al. (2017)
Coronastrum ellipsoideum	no	–	–	GQ507370	GQ507370	Pitsch et al. (2017)
Crucigenia lauterbornii	no	–	–	JQ356710	JQ356710	Pitsch et al. (2017)
Dicloster acuatus	no	–	–	FM205848	FM205848	Pitsch et al. (2017)
Dictyosphaerium ehrenbergianum	no	–	–	GQ487192	GQ487192	Pitsch et al. (2017)
Dictyosphaerium lacustre	no	–	–	GQ487204	GQ487204	Pitsch et al. (2017)
Dictyosphaerium libertatis	no	–	–	GQ487211	GQ487211	Pitsch et al. (2017)
Didymogenes anomala	no	–	–	FM205839	FM205839	Pitsch et al. (2017)
Didymogenes palatina	no	–	–	FM205840	FM205840	Pitsch et al. (2017)
Didymogenes soliella	no	–	–	AB731605	AB731605	Pitsch et al. (2017)
Didymogenes sphaerica	no	–	–	AB731603	AB731603	Pitsch et al. (2017)
Edaphochlorella mirabilis S2	no	–	–	OM472009	OM472009	Darienko et al. (2019)
Elliptochloris bilobata	no	–	–	HG972969	HG972969	Darienko et al. (2019)
Eremosphaera viridis	no	–	–	KY006556	KY006556	Darienko et al. (2019)
Franceia amphitricha	no	–	–	KM020072	KM020072	Darienko et al. (2019)
Hegewaldia parvula	no	–	–	FM205842	FM205842	Pitsch et al. (2017)
Heynigia dictyosphaerioides	no	–	–	GQ487221	GQ487221	Pitsch et al. (2017)
Heynigia riparia	no	–	–	GQ487225	GQ487225	Pitsch et al. (2017)
Hindakia fallax	no	–	–	GQ487223	GQ487223	Pitsch et al. (2017)

Taxon	Symbiotic or epiphytic strain	Host organism	Higher taxonomic group of host	GenBank accession number		Reference
				18S	ITS-5.8S-28S	
Hindakia tetrachotoma	no	–	–	GQ487238	GQ487238	Pitsch et al. (2017)
Jaagichlorella roystonensis	no	–	–	MH780940	MH780940	Darienko and Pröschold (2019)
Jaagichlorella africana	no	–	–	MH780938	MH780938	Darienko and Pröschold (2019)
Jaagichlorella geometrica TchVO	yes	*Trichodina chlorophora*	Ciliophora; Oligohymenophorea	PX870621	PX870621	Present study
Jaagichlorella geometrica	no	–	–	MH780944	MH780944	Darienko and Pröschold (2019)
Jaagichlorella hainangensis	no	–	–	MH780943	MH780943	Darienko and Pröschold (2019)
Jaagichlorella luteoviridis	no	–	–	MH780927	MH780927	Darienko and Pröschold (2019)
Jaagichlorella roystonensis	no	–	–	JN003601	–	Darienko and Pröschold (2019)
Jaagichlorella sphaerica	no	–	–	MH780945	MH780945	Darienko and Pröschold (2019)
Kalenjinia gelatinosa	no	–	–	GQ477061	GQ477061	Pitsch et al. (2017)
Kalinella apyrenoidosa	no	–	–	MH780947	MH780947	Darienko and Pröschold (2019)
Kalinella bambusicola	no	–	–	MH780946	MH780946	Darienko and Pröschold (2019)
Lobosphaera incisa	no	–	–	KM020046	KM020046	Darienko et al. (2019)
Lobosphaeropsis lobophora	no	–	–	FM205833	FM205833	Luo et al. (2010); Pitsch et al. (2017)
Marasphaerium gattermannii	no	–	–	GQ477057	GQ477057	Pitsch et al. (2017)
Masaia oloidia	no	–	–	GQ477059	GQ477059	Pitsch et al. (2017)
Meyerella planktonica	no	–	–	AY195973	AY543044	Fawley et al. (2005)
Meyerella sp.	yes	*Paramecium chlorelligerum*	Ciliophora; Oligohymenophorea	JX010741	–	Kreutz et al. (2012)
Micractinium belenophorum	no	–	–	FM205880	FM205880	Pitsch et al. (2017)
Micractinium conductrix	no	–	–	FM205852	FM205852	Pitsch et al. (2017)
Micractinium inermum	no	–	–	KF597304	KF597304	Pitsch et al. (2017)
Micractinium pusillum pop.1	no	–	–	FM205875	FM205875	Pitsch et al. (2017)
Micractinium pusillum pop.2	no	–	–	AF364101	FM205866	Serra et al. (2021)
Micractinium pusillum pop.3	no	–	–	AF364102	FM205838	Serra et al. (2021)
Micractinium reisseri pop.1	yes	*Paramecium bursaria*	Ciliophora; Oligohymenophorea	AB437244	AB437244	Serra et al. (2021)
Micractinium reisseri pop.2	yes	*Paramecium bursaria*	Ciliophora; Oligohymenophorea	AB506070	AB506070	Serra et al. (2021)
Micractinium sp.1	yes	*Paramecium bursaria*	Ciliophora; Oligohymenophorea	FM205851	FM205851	Luo et al. (2010)
Micractinium sp.2	yes	*Tetrahymena utriculariae*	Ciliophora; Oligohymenophorea	LT605003	LT605003	Pitsch et al. (2017)

Taxon	Symbiotic or epiphytic strain	Host organism	Higher taxonomic group of host	GenBank accession number		Reference
				18S	ITS-5.8S-28S	
Mucidosphaerium palustre	no	–	–	GQ487197	GQ487197	Pitsch et al. (2017)
Mucidosphaerium planctonicum	no	–	–	GQ487201	GQ487201	Pitsch et al. (2017)
Mucidosphaerium pulchellum	no	–	–	GQ487198	GQ487198	Pitsch et al. (2017)
Mucidosphaerium sphagnale	no	–	–	GQ487219	GQ487219	Pitsch et al. (2017)
Muriella terrestris	no	–	–	OM472001	–	Darienko et al. (2019)
Neocystis brevis	no	–	–	PQ276650	PQ276650	Darienko et al. (2019)
Oocystis heteromucosa	no	–	–	KY013466	KY013466	Darienko et al. (2019)
Parachlorella beijerinckii	no	–	–	FM205845	FM205845	Pitsch et al. (2017)
Parachlorella hussii	no	–	–	HM126550	HM126550	Pitsch et al. (2017)
Parachlorella kessleri	no	–	–	FM205846	FM205846	Pitsch et al. (2017)
Parachlorella kessleri CCAP 211/11G	no	–	–	FM205885	FM205885	Luo et al. (2010)
Parietochloris pseudoalveolaris	no	–	–	MT735204	MT735204	Darienko et al. (2019)
Pseudochlorella pyrenoidosa	no	–	–	LT560357	MW077556	Darienko et al. (2019)
Pumiliosphaera acidophila	no	–	–	LN610705	LN610705	Darienko et al. (2019)
Stichococcus bacillaris	no	–	–	FR717539	FR717539	Darienko et al. (2019)
Trebouxia asymmetrica	no	–	–	Z21553	AJ249565	Darienko et al. (2019)
Trebouxia impressa	no	–	–	Z21551	AF345891	Darienko et al. (2019)
Viridiella fridericiana	no	–	–	FM958481	FM958481	Darienko et al. (2019)
Watanabea reniformis	no	–	–	FM958480	FM958480	Darienko et al. (2019)

[a] *Chlorella vulgaris* pop.3 could not be identified by Hoshina et al. (2004)
A dash (–) indicates unavailable data

References

Darienko T, Pröschold T (2019) The genus *Jaagichlorella* Reisigl (Trebouxiophyceae, Chlorophyta) and its close relatives: an evolutionary puzzle. Phytotaxa 388:47–68. https://doi.org/10.11646/phytotaxa.388.1.2

Darienko T, Rad-Menéndez C, Campbell C, Pröschold T (2019) Are there any true marine Chlorella species? Molecular phylogenetic assessment and ecology of marine *Chlorella*-like organisms, including a description of *Droopiella* gen. nov. Syst Biodivers 17:811–829. https://doi.org/10.1080/14772000.2019.1690597

Fawley MW, Fawley KP, Owen HA (2005) Diversity and ecology of small coccoid green algae from Lake Itasca, Minnesota, USA, including *Meyerella planktonica*, gen. et sp. nov. (Trebouxiophyceae, Chlorophyta). Phycologia 44:35–48. https://doi.org/ 10.2216/0031-8884(2005)44[35:daeosc]2.0.co;2

Hoshina R, Imamura N (2008) Multiple origins of the symbioses in *Paramecium bursaria*. Protist 159:53–63. https://doi.org/10.1016/j.protis.2007.08.002

Hoshina R, Kusuoka Y (2016) DNA analysis of algal endosymbionts of ciliates reveals the state of algal integration and the surprising specificity of the symbiosis. Protist 167:174–184. http://dx.doi.org/10.1016/j.protis.2016.02.004

Hoshina R, Kamako S-I, Imamura N (2004) Phylogenetic position of endosymbiotic green algae in *Paramecium bursaria* Ehrenberg from Japan. Plant Biol 6:447–453. https://doi.org/ 10.1055/s-2004-820888

Kawaida H, Ohba K, Koutake Y, Shimizu H, Tachida H, Kobayakawa Y (2013) Symbiosis between *hydra* and *chlorella*: molecular phylogenetic analysis and experimental study provide insight into its origin and evolution. Mol Phylogenet Evol 66:906–914. https://doi.org/10.1016/j.ympev.2012.11.018

Kreutz M, Stoeck T, Foissner W (2012) Morphological and molecular characterization of *Paramecium* (Viridoparamecium nov. subgen.) *chlorelligerum* Kahl (Ciliophora). J Eukaryot Microbiol 59:548–563. https://doi.org/10.1111/j.1550-7408.2012.00638.x

Luo W, Pröschold T, Bock C, Krienitz L (2010) Generic concept in *Chlorella*-related coccoid green algae (Chlorophyta, Trebouxiophyceae). Plant Biol 12:545–553. https://doi.org/10.1111/j.1438-8677.2009.00221.x

Pitsch G, Adamec L, Dirren S, Nitsche F, Šimek K, Sirová D, Posch T (2017) The green *Tetrahymena utriculariae* n. sp. (Ciliophora, Oligohymenophorea) with its endosymbiotic algae (*Micractinium* sp.), living in traps of a carnivorous aquatic plant. J Eukaryot Microbiol 64:322–335. https://doi.org/ 10.1111/jeu.12369

Serra V, D'Alessandro A, Nitla V, Gammuto L, Modeo L, Petroni G, Fokin SI (2021) The neotypification of *Frontonia vernalis* (Ehrenberg, 1833) Ehrenberg, 1838 and the description of *Frontonia paravernalis* sp. nov. trigger a critical revision of frontoniid systematics. BMC Zool 6:e4. https://doi.org/10.1186/s40850-021-00067-9

Summerer M, Sonntag B, Sommaruga R (2008) Ciliate-symbiont specificity of freshwater endosymbiotic *Chlorella* (Trebouxiophyceae, Chlorophyta). J Phycol 44:77–84. https://doi.org/10.1111/j.1529-8817.2007.00455.x

Škaloud P, Steinová J, Řídká T, Vančurová L, Peksa O (2015) Assembling the challenging puzzle of algal biodiversity: species delimitation within the genus *Asterochloris* (Trebouxiophyceae, Chlorophyta). J Phycol 51:507–527. https://doi.org/10.1111/jpy.12295

Supplementary Table S4

Characterization and evolutionary models selected under the Bayesian information criterion by the IQTREE program for the four datasets analyzed

Dataset[a]	Marker	No. of chars	No. of taxa	Model[b]	A	C	G	T	[AC]	[AG]	[AT]	[CG]	[CT]	[GT]	I	Γ
#1	18S	1680	65	TIM2	0.2636	0.2087	0.2783	0.2494	1.5358	3.0110	1.5358	1.0000	5.2353	1.0000	0.4833	0.5097
#2	18S	1730	43	K2P	0.2500	0.2500	0.2500	0.2500	1.0000	3.3757	1.0000	1.0000	3.3757	1.0000	0.7707	–
	ITS region-28S	902	43	TNe	0.2500	0.2500	0.2500	0.2500	1.0000	2.9566	1.0000	1.0000	5.0643	1.0000	–	0.2234
	16S	887	43	TPM3	0.3410	0.1375	0.1992	0.3224	0.3758	2.7669	1.0000	0.3758	2.7669	1.0000	–	0.5004
	COI	652	43	K3Pu	0.3076	0.1248	0.1594	0.4082	5.2859	1.9706	1.0000	1.0000	1.9706	5.2859	–	0.4040
#3	18S	5738	130	TNe	0.2500	0.2500	0.2500	0.2500	1.0000	2.4812	1.0000	1.0000	4.2259	1.0000	0.2870	0.4399
#4	18S	5738	109	TN	0.2500	0.2500	0.2500	0.2500	1.0000	2.5446	1.0000	1.0000	4.14114	1.0000	0.2915	0.3776
	ITS region-28S	1929	109	GTR	0.2043	0.3078	0.2772	0.2107	0.9699	1.4178	0.8840	0.6204	3.0842	1.0000	0.0498	0.6561

[a] The first dataset comprised mobilid 18S rDNA sequences, the second dataset comprised mobilid nuclear (18S, ITS-5.8S-28S) and mitochondrial (16S and COI) sequences, the third dataset comprised algal 18S rDNA sequences, and the fourth dataset comprised algal 18S and ITS-5.8S-28S rDNA sequences

[b] Base frequencies (A, C, G, T), rate substitution matrix ([AC], [AG], [AT], [CG], [CT], [GT]), proportion of invariable sites (I), gamma distribution shape parameter (Γ)
